# Pedigree- and SNP-Associated Genetics and Recent Environment are the Major Contributors to Anthropometric and Cardiometabolic Trait Variation

**DOI:** 10.1371/journal.pgen.1005804

**Published:** 2016-02-02

**Authors:** Charley Xia, Carmen Amador, Jennifer Huffman, Holly Trochet, Archie Campbell, David Porteous, Generation Scotland, Nicholas D. Hastie, Caroline Hayward, Veronique Vitart, Pau Navarro, Chris S. Haley

**Affiliations:** 1 MRC Human Genetics Unit, MRC Institute of Genetics and Molecular Medicine, University of Edinburgh, Western General Hospital, Edinburgh, United Kingdom; 2 Generation Scotland, Centre for Genomic and Experimental Medicine, MRC Institute of Genetics and Molecular Medicine, University of Edinburgh, Western General Hospital, Edinburgh, United Kingdom; 3 A collaboration between the University Medical School and NHS in Aberdeen, Dundee, Edinburgh and Glasgow, Scotland, United Kingdom; 4 The Roslin Institute and R(D)SVS, University of Edinburgh, Midlothian, United Kingdom; Georgia Institute of Technology, UNITED STATES

## Abstract

Genome-wide association studies have successfully identified thousands of loci for a range of human complex traits and diseases. The proportion of phenotypic variance explained by significant associations is, however, limited. Given the same dense SNP panels, mixed model analyses capture a greater proportion of phenotypic variance than single SNP analyses but the total is generally still less than the genetic variance estimated from pedigree studies. Combining information from pedigree relationships and SNPs, we examined 16 complex anthropometric and cardiometabolic traits in a Scottish family-based cohort comprising up to 20,000 individuals genotyped for ~520,000 common autosomal SNPs. The inclusion of related individuals provides the opportunity to also estimate the genetic variance associated with pedigree as well as the effects of common family environment. Trait variation was partitioned into SNP-associated and pedigree-associated genetic variation, shared nuclear family environment, shared couple (partner) environment and shared full-sibling environment. Results demonstrate that trait heritabilities vary widely but, on average across traits, SNP-associated and pedigree-associated genetic effects each explain around half the genetic variance. For most traits the recently-shared environment of couples is also significant, accounting for ~11% of the phenotypic variance on average. On the other hand, the environment shared largely in the past by members of a nuclear family or by full-siblings, has a more limited impact. Our findings point to appropriate models to use in future studies as pedigree-associated genetic effects and couple environmental effects have seldom been taken into account in genotype-based analyses. Appropriate description of the trait variation could help understand causes of intra-individual variation and in the detection of contributing loci and environmental factors.

## Author Summary

Unravelling overall trait architecture of complex traits and diseases is important for phenotype prediction and disease prevention and correct modelling of the trait will further aid discovery of causative loci. Here we take advantage of genome-wide data and a large family-based study to examine the role of common genetic variants, pedigree-associated genetic variants, shared family environment, shared couple environment and shared sibling environment on 16 anthropometric and cardiometabolic traits. By analysing up to ~20,000 Scottish individuals, we find that common genetic variants, pedigree-associated genetic variants and recently-shared environment of couples are the most important contributors to variation in these traits, while past family and sibling environment have a limited impact. Further studies on the pedigree-associated genetic variation and the shared couple environment effect are needed, as little research has been devoted to them so far.

## Introduction

Phenotypic variation for a quantitative trait is attributable to the summed effects of genetic and environmental influences together with any covariances and interactions. The proportion of phenotypic variance contributed by genetic variation is termed the heritability (*h*^2^) [1]. The heritability scales the influence of genetic and environmental factors on phenotypic variation. This provides us with insights into the genetic and environmental architecture of human complex traits and our potential ability to dissect out loci associated with trait variation and is also useful for the prediction of heritable disease risk [2,3]. As a consequence, such knowledge is of potential value for clinical diagnosis, therapy, prevention and prognosis [4]. Therefore, obtaining unbiased estimates of variation caused by different factors and the heritability of traits relevant to health and disease processes is important.

A classic approach to gauging the heritability in humans is by comparing the observed phenotypic similarity to the expected genetic resemblance between relatives inferred from family pedigrees [5]. This method evaluates the pedigree based heritability ($h_{ped}^{2}$) indirectly without requiring information on the inheritance of individual loci and thus, is quite practical and still widely-used in twin, family and other pedigree studies [6,7]. Note that, $h_{ped}^{2}$ is often considered to be an estimate of the true heritability *h*^2^. Genome-wide association studies (GWAS), on the contrary, identify causal loci through their association with recorded genetic markers and then aggregate the proportion of variance explained by statistically-significant variants [8,9], which is sometimes referred to as the “GWAS heritability” ($h_{GWAS}^{2}$). Each approach has its limitations and drawbacks. Pedigree studies require genealogical information from known relatives to deduce their expected genetic resemblance and $h_{ped}^{2}$ may be biased due to the factors shared among relatives (including dominance, epistasis, common environment, genetic-by-environment correlation and genetic-by- environment interaction) if such effects are present and the available pedigree structure does not allow these to be accounted for in the analysis [10–12]. Although GWAS have been very successful at discovering novel loci for a range of polygenic disease and complex traits, they have been less successful at capturing the full extent of known trait genetic variance [11,12]. This is probably because of their failure to detect particular types of variants such as common variants with small effects, rare variants, copy number variants and structural variants, as a consequence of inadequate sample size, genotyping platform design and analyses used, together with the stringent statistical tests applied [10,13,14]. As a result, there usually is a substantial gap between the estimates of $h_{ped}^{2}$ and $h_{GWAS}^{2}$, often termed the “missing heritability” [11,15].

Recently, Yang *et al*. [16,17] have championed an approach, known as GREML [18], to estimate the amount of trait variance explained by SNPs. The estimation of the SNP (or genomic) heritability ($h_{g}^{2}$), which refers to the additive genetic effects captured by genotyped SNPs, utilises a matrix comprising realised genetic relationships inferred from genomic marker data originally gathered for GWAS (known as genomic relationship matrix or GRM) [16,17]. The $h_{g}^{2}$ estimate from this approach, when estimated using unrelated individuals, lies between the $h_{ped}^{2}$ and $h_{GWAS}^{2}$ estimates, and has been considered as a lower limit for the former and an upper limit for the latter [11,12]. As an example, for height, $h_{GWAS}^{2},\;h_{g}^{2}$ and $h_{ped}^{2}$ from three different studies are 0.10, 0.45 and 0.80 respectively [5,8,17]. This suggests that a substantial proportion of the genetic contribution to trait variation is SNP-associated and hence contributes to $h_{g}^{2}$ but not all this variation is detected by current GWAS, probably due to a combination of insufficient sample size and stringent significant thresholds employed. The difference between $h_{g}^{2}$ and $h_{ped}^{2}$ may be largely due to trait associated variants not in linkage disequilibrium (LD) with genotyped SNPs, such as rare variants, copy number variants (CNV) and other structural variants as mentioned above. Variation associated with such effects is captured by $h_{ped}^{2}$ due to strong LD in relatives [19].

Recent studies have started dissecting the heritable component of variation and other components shared among relatives by studying more complex populations made-up of both unrelated individuals and extended pedigrees [11,12,19]. For instance, Zaitlen *et al*. [12] have demonstrated that simultaneously including in a GREML analysis a GRM and a modified GRM (in which entries smaller than a certain threshold in the GRM are set to zero) can be used to jointly estimate SNP-associated and total heritabilities in the presence of relatives. We also note that shared environment may be an important contributor to heritability inflation when close relatives are included in analysis.

In this study, we use data from a single homogeneous cohort consisting of approximately 20,000 adults with varying degrees of relationships sampled from Scotland. The individuals have data on over 520,000 SNPs distributed across the autosomes. The dense marker information together with extended genealogical information allows us to partition the phenotypic variance and explore the genetic and environmental effects shared among related individuals (both biological relatives and couples).

We analyse eight anthropometric traits, comprising height, weight, fat, body mass index (BMI), hips, waist, waist-to-hips ratio (WHR) and a body shape index (ABSI) [20] and eight cardiometabolic traits, comprising levels of creatinine, urea, total cholesterol (TC) and high density lipoprotein (HDL) in serum, level of glucose in blood, systolic blood pressure (SBP), diastolic blood pressure (DBP) and heart rate (HR).

In our work, we implement alternative models to estimate effects that might contribute to the variation in the 16 traits analysed. Results show that, with these data, we can separate total genetic variation into SNP-associated and pedigree-associated genetic influences. We also observe that past family environment and shared full-sibling environment generally have a limited impact on trait variation, whereas the effect in couples of living in the current (shared) environment is always important in our data.

## Results

### Overview of the methods

We conducted variance component analyses to dissect the phenotypic variation for traits recorded in the Generation Scotland: Scottish Family Health Study (GS:SFHS) cohort [21] into genetic and environmental factors. Analyses utilised a mixed-model approach implemented in a restricted maximum likelihood (REML) framework using the GCTA software [16]. The population was divided into two tranches of approximately equal size and genotyped in two stages. All initial analyses were performed with the first 10,000 genotyped individuals, (named GS10K). GS10K comprised small nuclear families (largely two parents and two offspring) together with unrelated individuals, although inevitably there were second degree and more distant relationships included. The second tranche completed the genotyping of the rest of the population (another 10,000 individuals) including further relatives in incomplete families (e.g. missing samples from parents and additional siblings, as well as other relationships), resulting particularly in a proportional increase in the number of second and third degree relationships (Table 1). To confirm results obtained from GS10K, some of the analyses were repeated in the whole 20,000 individual sample (named GS20K).

**Table 1:** Comparisons of sample sizes, number of non-zero off-diagonal entries and number of pairwise relationships of different degrees between GS10K and GS20K.

	**GS10K**	**GS20K**	**Ratio**
**No. IDs**	9,863	20,032	1: 2.03
**Matrix**	**No. Non-Zero Off-diagonal Entries** ^a^		**Ratio**
G	48,634,453 ^b^	200,630,496	1: 4.13
K	8,080	41,174	1: 5.10
F	4,821	20,115	1: 4.17
C	1,283	1,767	1: 1.38
S	676	8,495	1: 12.57
**Degree of Relationship** ^**c**^	**No. Pairs**		**Ratio**
1^st^ degree relatives	3,529	18,320	1: 5.19
2^nd^ degree relatives	441	7,851	1: 17.80
3^rd^ degree relatives	500	4,129	1: 8.26
4^th^ degree relatives	1,099	3,950	1: 3.59
5^th^ degree relatives	3,891	11,032	1: 2.84
unrelated individuals	48,624,993	200,585,162	1: 4.13

^a^ The number of off-diagonal entries is calculated in the lower triangular part of all the matrices

^b^ For matrix G, all the off-diagonal entries are different from zero, so the value represents the total number of off-diagonal entries

^c^ Distance of relationship is identified according to an approximate range of the expected pair-wise relatedness, 0.5^i-0.5^ to 0.5^i+0.5^ for *i*^th^ degree relatives.

**Fig 1:**
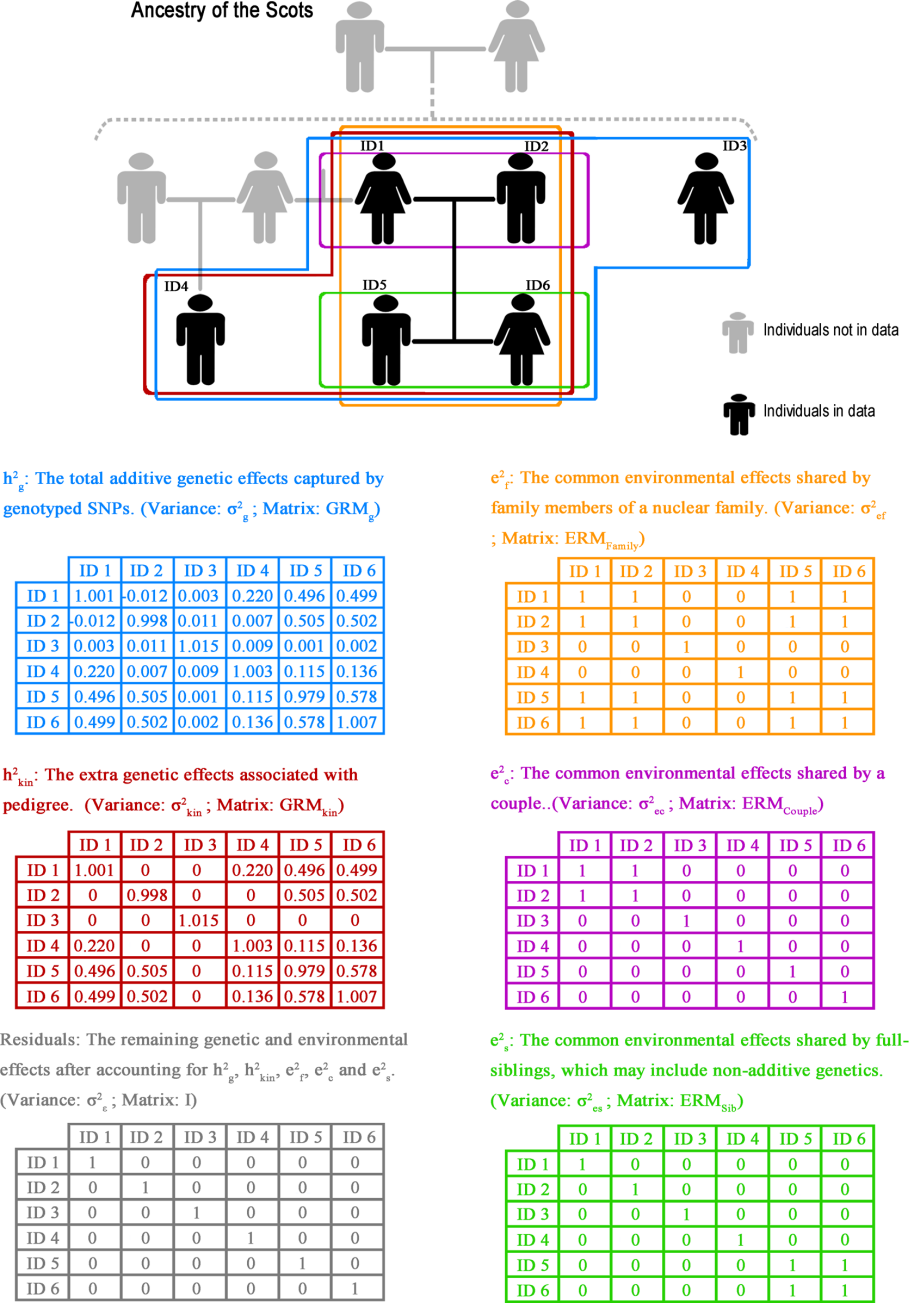
Illustration of the model and matrices. The diagram shows the relationship between the tested genetic/environmental effect and the individuals in an example pedigree. Each colour represents a specific effect and individuals affected by that effect are circled with that colour. People in grey or black are the people not in or in the data. Examples of how the relationship matrices for those effects look are also given.

We first explored the extent to which estimates of $h_{g}^{2}$ were inflated by the inclusion of relatives. We subsequently analysed our data allowing trait variation to be potentially influenced by both genetic and environmental effects. We assumed that the genetic effects comprised additive genetic effects associated with genotyped SNPs ($h_{g}^{2}$) and additional additive genetic effects associated with pedigree but not with genotyped SNPs ($h_{kin}^{2}$), and we assumed that the environmental effects potentially comprised nuclear family effects ($e_{f}^{2}$) common to both parents and offspring, full-sibling effects ($e_{s}^{2}$) common to just siblings and couple effects ($e_{c}^{2}$) common to just the members of a couple (Fig 1). The total heritability, termed $h_{gkin}^{2}$ in this manuscript, referred to as $h_{IBS>t_{*}}^{2}$ in Zaitlen *et al*. [12] and comparable to $h_{ped}^{2}$ from traditional pedigree studies, was estimated as the sum of $h_{g}^{2}$ and $h_{kin}^{2}$ for each model. To allow estimation of the influence of each effect, we generated five design matrices: **GRM**_**g**_, **GRM**_**kin**_, **ERM**_**Family**_, **ERM**_**Sib**_ and **ERM**_**Couple**_ respectively, where GRM refers to genomic relationship matrices and ERM refers to environmental relationship matrices.

For brevity, we named different alternative models using abbreviations according to first subscript letter of the effects examined. We coded ‘**G**’ for **GRM**_**g**_, ‘**K**’ for **GRM**_**kin**_, ‘**F**’ for **ERM**_**Family**_, ‘**S**’ for **ERM**_**Sib**_ and ‘**C**’ for **ERM**_**Couple**_–e.g. model ‘**GKC**’ = **GRM**_**g**_ + **GRM**_**kin**_ + **ERM**_**Couple**_. All models included a residual matrix (allowing effects specific to an individual that were not shared with any other member of the population).

We identified the most appropriate model for each trait by a stepwise model selection process via removing non-significant components from the full model based on a Wald test of their estimated effect and a likelihood ratio test (LRT), and we estimated the effects of significant factors using the selected models in GS10K. We repeated the model selection and corresponding variance component analyses in GS20K to identify differences resulting from analysing a more complex population structure, encompassing a larger proportion of close relationships.

More details about traits, matrices and models are given in Material and Methods and S1 Table and S2 Table. In the main manuscript, we only list results for the final models identified by the model selection procedure and the full model, but a comprehensive list of estimates obtained for the different effects for each trait and each model is available in S3 Table and S4 Table.

Model robustness and the effectiveness of the model selection were tested using simulated data based on GS10K.

### Simulation study: Robustness of the models

We conducted a simulation study using real genotype and pedigree information from GS10K to evaluate the robustness of our models. To make computation feasible, we mainly focused on data simulated under the simplest and most complex models (models ‘**G**’, ‘**K**’, ‘**F**’, ‘**S**’, ‘**C**’, ‘**GK**’, ‘**GF**’ and ‘**GKFSC**’) and those representing the commonest conclusions of model selection in analyses of the real GS10K data (models ‘**GF**’, ‘**GFS**’, ‘**GKC**’ and ‘**GKSC**’). S5 Table shows the simulated and observed values for each parameter as well as the model we used for analyses in different scenarios.

In the first scenario, we examined the performance of our models (models ‘**G**’, ‘**K**’, ‘**F**’, ‘**S**’ and ‘**C**’) when simulated phenotypes were only contributed by one of the five corresponding effects plus residual variation. Under these models (S5 Table), the mean of overall estimates per parameter was very close to its simulated value, indicating that our design matrices **GRM**_**g**_**, GRM**_**kin**_, **ERM**_**Family**_, **ERM**_**Couple**_ and **ERM**_**Sib**_ worked well in simple models and were able to capture their corresponding effects even when the simulated variance associated with an effect was low (≤ 3%).

In the second scenario, we evaluated the performance of our models (models ‘**GK**’ and ‘**GF**’) when the simulated phenotypes were determined by SNP-associated genetic effects and one of the familial effects (either pedigree-associated genetics or nuclear family environment) plus residual variation. Results (S5 Table) indicate that, in cohort with familial structure, failure to account for or inaccurate modelling of familial effects (i.e. when models used were inconsistent with phenotypic contributors) would result in upward bias for $h_{g}^{2}$ in the presence of relatives. However, this upward bias due to the confounding familial factors could be eliminated by either excluding nominally related individuals or using the appropriate models for analysis. The former method removes the ability to estimate the familial effects as well as reducing the sample size, whereas using the appropriate models, estimates obtained were very close to their parameter settings and gave a good idea of the magnitude and approximate values of SNP and familial effects as well as the total proportion of variance explained by additive genetics ($h_{gkin}^{2}=h_{g}^{2}+h_{kin}^{2}$), despite the fact that the means of estimates of $h_{g}^{2},\;h_{kin}^{2}$ and $e_{f}^{2}$ were usually significantly different from the original parameter settings.

In the third scenario, we inspected the performance of the full model ‘**GKFSC**’ and models selected from analyses of real phenotypes in GS10K other than ‘**GF**’ (models ‘**GFS**’, ‘**GKC**’ and ‘**GKSC**’). Results (S5 Table) demonstrate that all models were robust in terms of the mean of overall estimates per parameter being either unbiased or very close to original settings.

**Fig 2:**
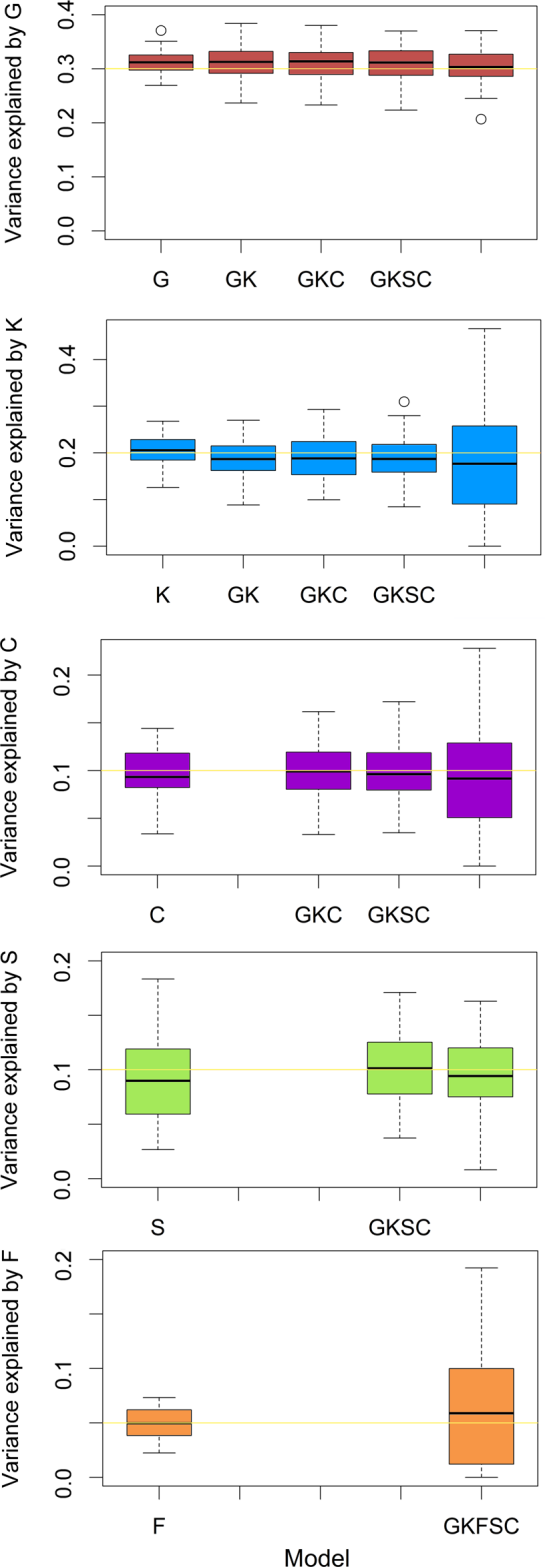
Boxplots for estimates of each component obtained from models ‘G’, ‘K’, ‘F’, ‘S’, ‘C’, ‘GK’, ‘GKC’, ‘GKSC’ and ‘GKFSC’. X-axis: the contributors to the simulated phenotype and the model used (matched model); Y-axis: proportion of total phenotypic variance captured by each design matrix. Yellow lines: simulated value for each component. Parameter settings: $h_{g}^{2}$ = 0.3, $h_{kin}^{2}$ = 0.2, $e_{c}^{2}$ = 0.1, $e_{s}^{2}$ = 0.1 and $e_{f}^{2}$ = 0.05. For example, the 2^nd^ boxplot of the 3^rd^ graph means that, the simulated phenotypes are contributed by 30%, 20%, 10% and 40% of SNP-associated, pedigree-associated, couple environmental and residual effects respectively; we conducted variance component analyses for all replicates using the matched model ‘**GKC**’ and the estimates of $e_{c}^{2}$ range from about 8% to 12% with a mean of 10%, as expected.

Fig 2 summarizes the main results from these simulations, showing the overall performance of our design matrices from simple models to complex models. The median of estimates for each component was unbiased across simple and complex models, however, the estimates for $h_{kin}^{2},\;e_{f}^{2}$ and $e_{c}^{2}$ were quite variable in the full model, probably due to limitations imposed by the data structure. All of the above verify the robustness of our models.

### Simulation study: Effectiveness of the model selection procedure

Although we confirmed that our models were robust (S5 Table and Fig 2), the potentially high correlation between **ERM**_**Family**_ matrix and combined **ERM**_**Couple**_ and **GRM**_**kin**_ matrices may make it challenging to jointly estimate $h_{kin}^{2},\;e_{f}^{2}$ and $e_{c}^{2}$ accurately in our sample as the standard errors for those parameter estimates obtained from the full model were high (S4 Table). Thus the most challenging part of our study may be to precisely dissect pedigree-associated genetic effects, shared nuclear family environment and shared couple environment. Therefore, we performed model selection using simulated data to test our model selection procedure where simulated phenotypes were contributed by moderate SNP-associated genetic effects and low sibling environmental effects plus a) moderate nuclear family environmental effects but low pedigree-associated genetic effects and couple environmental effects; b) low nuclear family environmental effects but moderate pedigree-associated genetic effects and couple environmental effects; or c) moderate nuclear family environmental effects, pedigree-associated genetic effects and couple environmental effects. All scenarios included residual variation.

S6 Table shows the parameter settings and the summary of model selection procedure performance for these scenarios. We expected that our model selection procedure was able to identify SNP genetics (**GRM**_**g**_) and nuclear family environment (**ERM**_**Family**_) or SNP and pedigree genetics (**GRM**_**kin**_) and couple environment (**ERM**_**Couple**_) or SNP and pedigree genetics and nuclear family and couple environment accordingly, since they were the major factors in each corresponding scenario.

As results demonstrated, in all situations our model selection procedure generally (≥80%) selected the appropriate model which contains all major components of phenotypic variation. The remaining times in the first two of these scenarios, pedigree-associated genetic effects or those plus shared couple environment were selected instead of nuclear family environmental effects or vice versa, and in the remaining two replicates in the third of these scenarios we missed pedigree-associated genetic effects. In addition, our model selection never fully detected all minor contributions to the phenotype in the first two of these scenarios when the minor effects were too small (e.g. effects contribute to ≤5% of the phenotypic variance).

Both issues identified above (~20% chance of selecting inappropriate models and failure to identify all minor effects) are likely to have been due to limitations in the data structure of GS10K, which provides too few of the appropriate relationships for corresponding effects (pedigree-associated genetics, nuclear family, sibling and couple environment) to resolve correlations between parameters and detect minor effects. These limitations have been greatly ameliorated in the GS20K data.

We also conducted variance component analyses using the final selected model for each replicate (S6 Table). For those replicates that had appropriate models after model selection, the estimates of factors that remained in the models were usually close to, and not significantly different from, their simulated values, indicating that the results from selected models were reliable. More details about simulation study can be found in S1 Text, S5 Table and S6 Table.

### Impact of inclusion of 1^st^ degree relatives on the genomic heritability in GS10K

In the first analyses of the real data, we looked for evidence of familial effects (either pedigree-associated genetics or nuclear family environment) in our cohort. As shown by simulation (S5 Table), if there were any familial effects, we should obtain inflated estimates of $h_{g}^{2}$ when we conducted variance component analyses using model **‘G’** in the presence of relatives, compared to the estimates of $h_{g}^{2}$ given from the unrelated subpopulation. GS10K consists of nearly 10,000 genotyped individuals with multiple degrees of relationship, which allows us to explore the impact of familial effects on $h_{g}^{2}$ estimation in this cohort.

Table 1 shows the population structure of genotyped individuals in GS10K. The degree of relationship between two individuals was identified according to an approximate range of the expected pair-wise relatedness (*r*), which was from 0.5^i-0.5^ to 0.5^i+0.5^ for *i*^th^ degree relatives (e.g. pairs of individuals with relatedness from 0.354 to 0.707 were considered as 1^st^ degree relatives).

With these criteria, GS10K consisted of more than 3,500 pairs of 1^st^ degree relatives, around 450 pairs of 2^nd^ and 500 pairs of 3^rd^ degree relatives, but the majority of pairs of individuals (over 99.9%) were genetically unrelated (more distant than 5^th^ degree relatives, *r* ≤ 0.022). In total, there were around 6,600 unrelated individuals (defined using the criteria described above) in GS10K.

**Fig 3:**
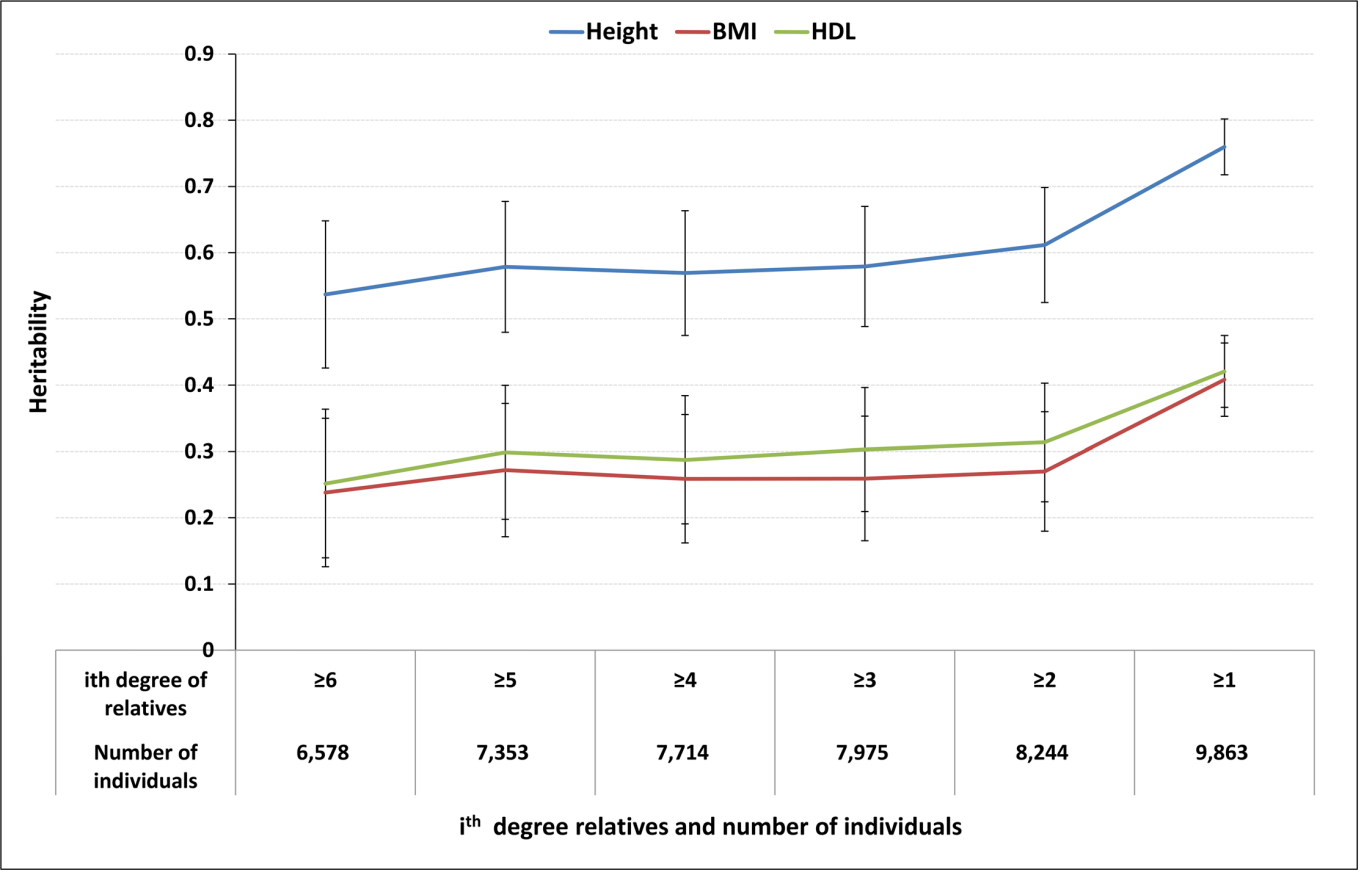
Heritability estimates using subpopulations of GS10K with different GRM cut-off points. X-axis: the number of individuals among whom the pairwise relationship is larger than a specified degree; Y-axis: Heritability estimates with ± 2 *s*.*e*.

We estimated $h_{g}^{2}$ for each trait using model ‘**G**’ for subpopulations of GS10K made-up of individuals with different degrees of relatedness (using the upper bound of the expected relatedness of each category as GRM cut-off points in GCTA). Fig 3 shows how $h_{g}^{2}$ estimates for height, BMI and HDL changed as we progressively included more closely related individuals in the relationship matrix. Results for the remaining traits are shown in S3 Table.

In general, $h_{g}^{2}$ estimates were stable as we gradually added more closely related individuals in the analyses until the inclusion of 1^st^ degree relatives that resulted in inflation of the estimates (Fig 3 and S3 Table). Based on our results, $h_{g}^{2}$ was overestimated only when 1^st^ degree relatives were included. For glucose and DBP, the $h_{g}^{2}$ estimates did not appear inflated after 1^st^ degree relatives were included, suggesting that these traits were not affected by familial effects (S3 Table).

### Variance component analyses using the full model ‘GKFSC’ and stepwise model selection in GS10K

The increase in $h_{g}^{2}$ estimates resulting from the inclusion of 1^st^ degree relatives provided evidence of familial variation in our cohort. However, it is not clear whether these familial effects are due to pedigree-associated genetic effects or shared nuclear family environment or both because either of them has the ability to inflate $h_{g}^{2}$ estimates (this was also observed in the simulation data: S5 Table: scenario ii). Therefore, we attempted to tease out this familial variance from the total phenotypic variance and dissect the familial variation as well as the remaining trait variation further using the full model ‘**GKFSC**’ and the stepwise selection procedure to define a final model containing the most important effects contributing to trait variation.

Table 2 shows the results for final models selected from stepwise model selection strategies and for the proportions of total phenotypic variance explained by different effects using final models, as well as for those obtained using the full model.

**Table 2:** Results of variance component analyses for anthropometric and cardiometabolic traits using final models selected from the stepwise model selection and the full model in GS10K.

Trait	Model		GRM_g_	GRM_kin_	ERM_Family_	ERM_Sib_	ERM_Couple_
			*h*^*2*^_*g*_ (*s*.*e*.)	*h*^*2*^_*kin*_ (*s*.*e*.)	*e*_*f*_^*2*^ (*s*.*e*.)	*e*_*s*_^*2*^ (*s*.*e*.)	*e*_*c*_^*2*^ (*s*.*e*.)
**Anthropometric Traits**							
Height	Selected	**GKC**	0.47(0.04)	0.36(0.05)			0.16(0.03)
	Full	**GKFSC**	0.45(0.04)	0.36(0.17)^NS^	0.00(0.08)^NS^	0.02(0.03)^NS^	0.17(0.09)^NS^
Weight	Selected	**GF**	0.28(0.03)		0.18(0.02)		
	Full	**GKFSC**	0.28(0.04)	0.17(0.17)^NS^	0.10(0.09)^NS^	0.01(0.04)^NS^	0.09(0.09)^NS^
Fat	Selected	**GKC**	0.26(0.04)	0.26(0.06)			0.19(0.03)
	Full	**GKFSC**	0.26(0.04)	0.21(0.18)^NS^	0.02(0.09)^NS^	0.02(0.04)^NS^	0.16(0.09)
BMI	Selected	**GKC**	0.25(0.04)	0.33(0.05)			0.21(0.03)
	Full	**GKFSC**	0.25(0.04)	0.19(0.17)^NS^	0.07(0.09)^NS^	0.00(0.04)^NS^	0.14(0.09)
Hips	Selected	**GKC**	0.21(0.04)	0.27(0.06)			0.17(0.03)
	Full	**GKFSC**	0.21(0.04)	0.18(0.18)^NS^	0.05(0.09)^NS^	0.00(0.04)^NS^	0.12(0.09)^NS^
Waist	Selected	**GKC**	0.16(0.04)	0.36(0.06)			0.20(0.03)
	Full	**GKFSC**	0.15(0.04)	0.44(0.17)^NS^	0.00(0.09)^NS^	0.00(0.04)^NS^	0.24(0.09)
WHR	Selected	**GKC**	0.15(0.04)	0.19(0.06)			0.09(0.03)
	Full	**GKFSC**	0.13(0.04)	0.29(0.17)^NS^	0.00(0.09)^NS^	0.00(0.04)^NS^	0.13(0.09)^NS^
ABSI	Selected	**GKC**	0.10(0.04)	0.19(0.06)			0.05(0.03)
	Full	**GKFSC**	0.08(0.04)	0.27(0.17)^NS^	0.00(0.08)^NS^	0.00(0.04)^NS^	0.08(0.09)^NS^
**Cardiometabolic Traits**							
Urea	Selected	**GF**	0.13(0.03)		0.10(0.02)		
	Full	**GKFSC**	0.15(0.04)	0.00(0.17)^NS^	0.08(0.09)^NS^	0.00(0.05)^NS^	0.04(0.09)
Creatinine	Selected	**GKSC**	0.24(0.04)	0.45(0.05)		0.07(0.03)	0.16(0.03)
	Full	**GKFSC**	0.24(0.04)	0.39(0.18)	0.03(0.09)^NS^	0.07(0.04)	0.13(0.09)^NS^
Glucose	Selected	**GC**	0.19(0.03)				0.05(0.03)
	Full	**GKFSC**	0.19(0.04)	0.00(0.17)^NS^	0.00(0.09)^NS^	0.09(0.05)^NS^	0.05(0.09)
TC	Selected	**GFS**	0.17(0.03)		0.09(0.02)	0.12(0.04)	
	Full	**GKFSC**	0.15(0.04)	0.12(0.18)^NS^	0.05(0.09)^NS^	0.12(0.04)	0.02(0.09)^NS^
HDL	Selected	**GKC**	0.30(0.04)	0.26(0.05)			0.15(0.03)
	Full	**GKFSC**	0.29(0.04)	0.35(0.16)^NS^	0.00(0.08)^NS^	0.01(0.04)^NS^	0.19(0.08)^NS^
SBP	Selected	**GKC**	0.15(0.04)	0.13(0.06)			0.10(0.03)
	Full	**GKFSC**	0.14(0.04)	0.18(0.18)^NS^	0.00(0.09)^NS^	0.08(0.05)^NS^	0.13(0.09)^NS^
DBP	Selected	**GC**	0.17(0.03)				0.09(0.03)
	Full	**GKFSC**	0.13(0.04)	0.00(0.18)^NS^	0.04(0.09)^NS^	0.03(0.05)^NS^	0.03(0.09)^NS^
HR	Selected	**GF**	0.14(0.03)		0.10(0.02)		
	Full	**GKFSC**	0.03(0.04)^NA^	0.00(0.18)^NS^	0.10(0.09)^NS^	0.00(0.05)^NS^	0.00(0.10)^NS^

^NS^ Not significant. That variance component is non-significant according to LRT with p-value > 0.05.

^NA^ Not available. Cannot test the significance of that variance component because the failure in the reduced model.

The mean estimates for $h_{g}^{2},\;h_{kin}^{2},\;e_{f}^{2},\;e_{s}^{2}$ and $e_{c}^{2}$ across all traits in the full model were 0.18, 0.22, 0.03, 0.03 and 0.11, respectively. However, the majority of estimates for parameters other than $h_{g}^{2}$ obtained using the full model were not significantly different from zero according to either the Wald test or LRT performed and had large standard errors in general. These results suggest that the full model ‘**GKFSC**’ may suffer from the inclusion of correlated factors, as foreseen in the simulation study, probably due to a low number of different types of pairwise relationship in GS10K.

Therefore, we utilised a model selection procedure designed to provide more precise estimates of the parameters retained in a more robust and parsimonious final model, where the least significant effects are removed from the model. More details about the selection procedure are given in Material and Methods. We have demonstrated the effectiveness of our model selection procedure by simulation in the previous section and S6 Table.

As shown in Table 2, SNP-associated genetic effects (represented by **GRM**_**g**_) were retained in the final models for all 16 traits, indicating that all traits examined here are heritable. Regarding variation associated with families, pedigree-associated genetic effects (represented by **GRM**_**kin**_) and nuclear family environmental effects (represented by **ERM**_**Family**_) were retained in the final models for 10 and 4 out of 16 traits respectively. However, in GS10K, the data structure did not allow for both familial effects to be retained together in the final models for any trait. Additionally, the final models for glucose and DBP included neither **GRM**_**kin**_ nor **ERM**_**Family**_, which is consistent with the previous conclusion derived from S3 Table, suggesting that familial effects may be limited for these traits.

The additional environmental influences of couple environmental effects (represented by **ERM**_**Couple**_) were retained in the final models for 12 out of 16 traits and sibling environmental effects (represented by **ERM**_**Sib**_) only remained for creatinine and TC.

Although the final model varied between traits, the model ‘**GKC**’ was most often selected (9 out of 16 traits) in the model selection procedure in GS10K. Therefore, this suggests that the common environment shared by couples, SNP-associated and pedigree-associated genetic effects are important for the control of a large proportion of the human complex traits we examined, while the shared family and full-sibling environment have a more limited impact

SNP-associated genetic effects (**GRM**_**g**_) in the final models provided estimates of $h_{g}^{2}$ ranging between 0.10 and 0.30 with a mean of 0.19 for the 15 traits, excepting height for which nearly half of its phenotypic variation (0.47) was SNP-associated.

For the 10 traits that retained pedigree-associated genetic effects (**GRM**_**kin**_) in the final models, the estimates of $h_{kin}^{2}$ ranged from 0.13 to 0.36 with a mean of 0.26, except for creatinine for which nearly half of its phenotypic variation (0.45) was pedigree-associated. For the 10 traits that retained both **GRM**_**g**_ and **GRM**_**kin**_ in the final models, the estimates of $h_{kin}^{2}$ accounted for 56% of the total heritability ($h_{gkin}^{2}=h_{g}^{2}+h_{kin}^{2}$).

Regarding nuclear family environmental effects, the estimates of $e_{f}^{2}$ for 4 traits that retained **ERM**_**Family**_ in the final models were of 18% for anthropometric and of 10% for cardiometabolic traits.

Creatinine and TC were the only two traits for which the common sibling environment (**ERM**_**Sib**_) was kept in the final models, and $e_{s}^{2}$ contributed 7% and 12% of their phenotypic variance respectively.

For those 12 traits that demonstrated evidence of couple effects (i.e. retained **ERM**_**Couple**_ in the final models), $e_{c}^{2}$ accounted for 13.5% of the phenotypic variance on average (of 15% for anthropometric traits and of 11% for cardiometabolic traits).

Compared to the results from the full model in Table 2, using the selected final models provided similar but more precise (i.e. with smaller standard errors) parameter estimates. Therefore, whereas the full models gave a general picture of the important components in the architecture of the traits, the final selected models provided a parsimonious model with more precise estimates of the most important effects.

### Results for model selection and corresponding variance component estimates in GS20K analyses

We added an extra 10,000 genotyped and phenotyped individuals from the same population, providing 20,000 individuals in total, in order to confirm and build upon the results of the model selection in a more complex data set. The difference in sample sizes and numbers of different relationships between GS10K and GS20K is shown in Table 1. The extra 10,000 genotyped individuals in GS20K consisted mainly of the relatives of those already genotyped in GS10K, which substantially increased the proportion of 2^nd^ and 3^rd^ degree and sibling relationships in GS20K. We repeated the model selection procedure and corresponding variance component analyses using selected models in GS20K to identify changes resulting from the increased complexity and sample size of the population.

Results for model selection and variance component analyses using the final selected model as well as the full model are shown in Table 3. In general, the parameter estimates obtained from the full model in GS20K were similar to those obtained from the full model in GS10K but the number of non-significant estimates were much lower due to smaller standard errors. Note that standard errors of estimates are not only reduced using GS20K, but, unlike results from GS10K in Table 2, are also similar between full and reduced models, suggesting the change is due to improved structure of the data to separate effects as well as increased sample size.

**Table 3:** Results of variance component analyses for anthropometric and cardiometabolic traits using final models selected from the stepwise model selection and the full model in GS20K.

Trait	Model		GRM_g_	GRM_kin_	ERM_Family_	ERM_Sib_	ERM_Couple_
			*h*^*2*^_*g*_ (*s*.*e*.)	*h*^*2*^_*kin*_ (*s*.*e*.)	*e*_*f*_^*2*^ (*s*.*e*.)	*e*_*s*_^*2*^ (*s*.*e*.)	*e*_*c*_^*2*^ (*s*.*e*.)
**Anthropometric Traits**							
Height	Selected	**GKFC**	0.43(0.02)	0.45(0.04)	0.01(0.02)		0.12(0.02)
	Full	**GKFSC**	0.43(0.02)	0.44(0.04)	0.01(0.02)	0.01(0.01)^NS^	0.11(0.02)^NS^
Weight	Selected	**GKFC**	0.27(0.02)	0.27(0.05)	0.05(0.02)		0.13(0.03)
	Full	**GKFSC**	0.27(0.02)	0.27(0.05)	0.04(0.02)^NS^	0.02(0.01)^NS^	0.13(0.03)
Fat	Selected	**GKSC**	0.24(0.02)	0.25(0.05)		0.04(0.01)	0.19(0.02)
	Full	**GKFSC**	0.24(0.02)	0.22(0.05)	0.02(0.02)^NS^	0.03(0.01)	0.17(0.03)
BMI	Selected	**GKFC**	0.25(0.02)	0.23(0.05)	0.05(0.02)		0.15(0.03)
	Full	**GKFSC**	0.25(0.02)	0.23(0.05)	0.04(0.02)	0.01(0.01)^NS^	0.15(0.03)
Hips	Selected	**GKFC**	0.22(0.02)	0.20(0.05)	0.05(0.02)		0.12(0.03)
	Full	**GKFSC**	0.22(0.02)	0.20(0.05)	0.05(0.03)	0.01(0.01)^NS^	0.12(0.03)
Waist	Selected	**GKSC**	0.19(0.02)	0.31(0.03)		0.04(0.01)	0.18(0.02)
	Full	**GKFSC**	0.19(0.02)	0.25(0.05)	0.03(0.02)^NS^	0.03(0.01)	0.15(0.03)
WHR	Selected	**GKSC**	0.11(0.02)	0.19(0.03)		0.04(0.02)	0.08(0.03)
	Full	**GKFSC**	0.11(0.02)	0.22(0.05)	0.00(0.03)^NS^	0.03(0.02)	0.09(0.03)
ABSI	Selected	**GKSC**	0.11(0.02)	0.21(0.03)		0.03(0.02)	0.05(0.03)
	Full	**GKFSC**	0.10(0.02)	0.24(0.05)	0.00(0.03)^NS^	0.02(0.02)^NS^	0.07(0.03)^NS^
**Cardiometabolic Traits**							
Urea	Selected	**GKSC**	0.15(0.02)	0.10(0.03)		0.03(0.02)	0.13(0.03)
	Full	**GKFSC**	0.14(0.02)	0.16(0.05)^NS^	0.00(0.03)^NS^	0.03(0.02)^NS^	0.16(0.03)
Creatinine	Selected	**GKSC**	0.23(0.02)	0.37(0.03)		0.07(0.01)	0.11(0.02)
	Full	**GKFSC**	0.21(0.02)	0.46(0.05)	0.00(0.02)^NS^	0.07(0.01)	0.14(0.03)^NS^
Glucose	Selected	**GSC**	0.17(0.02)			0.05(0.01)	0.05(0.02)
	Full	**GKFSC**	0.15(0.02)	0.00(0.05)^NS^	0.03(0.03)^NS^	0.04(0.02)	0.03(0.03)^NS^
TC	Selected	**GKSC**	0.19(0.02)	0.14(0.03)		0.07(0.02)	0.06(0.02)
	Full	**GKFSC**	0.19(0.02)	0.12(0.06)	0.01(0.03)^NS^	0.06(0.02)	0.05(0.03)^NS^
HDL	Selected	**GKSC**	0.27(0.02)	0.29(0.03)		0.03(0.01)	0.13(0.02)
	Full	**GKFSC**	0.27(0.02)	0.27(0.05)	0.01(0.02)^NS^	0.02(0.01)	0.11(0.03)
SBP	Selected	**GKSC**	0.12(0.02)	0.07(0.03)		0.07(0.02)	0.09(0.02)
	Full	**GKFSC**	0.12(0.02)	0.08(0.05)^NS^	0.00(0.03)^NS^	0.07(0.02)	0.09(0.03)
DBP	Selected	**GSC**	0.16(0.02)			0.08(0.01)	0.09(0.02)
	Full	**GKFSC**	0.14(0.02)	0.00(0.05)^NS^	0.02(0.03)^NS^	0.06(0.02)	0.07(0.03)
HR	Selected	**GKSC**	0.14(0.02)	0.12(0.03)		0.04(0.02)	0.07(0.02)
	Full	**GKFSC**	0.14(0.02)	0.10(0.05)	0.01(0.03)^NS^	0.04(0.02)	0.06(0.03)

^NS^ Not significant. That variance component is non-significant according to LRT with p-value > 0.05.

**Fig 4:**
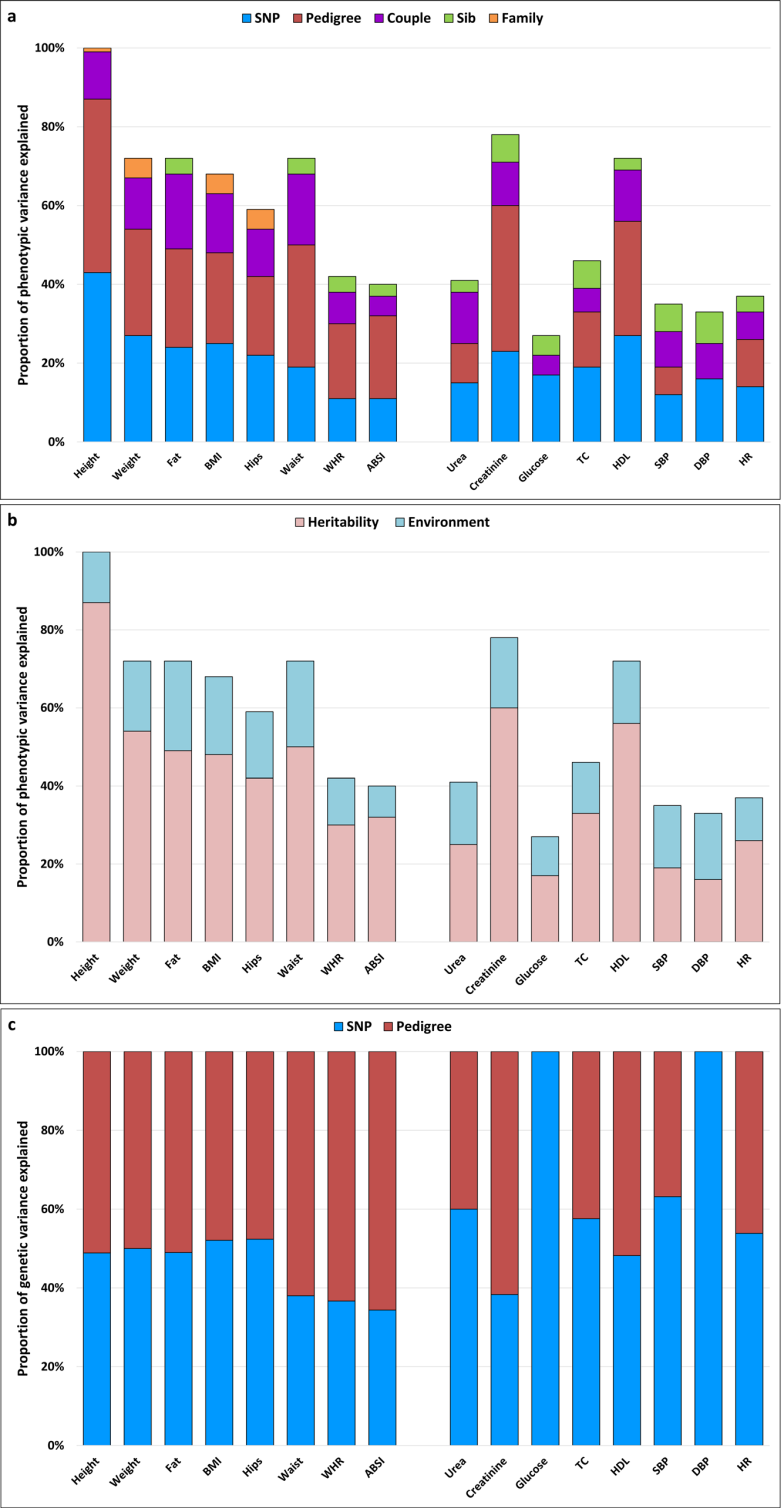
Results of variance component analysis using final selected models for anthropometric and cardiometabolic traits in GS20K. X-axis: names of phenotype; Y-axis: proportion of phenotypic/genetic variance explained by the different components. a) Proportion of phenotypic variance explained by genetics and environment for each trait. b) Proportion of phenotypic variance explained by different components kept in the selected model for each trait. c) Proportion of genetic variance explained by SNP-associated and pedigree-associated genetic effects.

The final models selected from model selection in GS20K were generally similar to those in GS10K, but, owing to the presence of more nuclear family members and siblings in GS20K, we now had better power to detect the past environmental effects (either nuclear family environment or sibling environment), although the estimated effects were usually small. Moreover, due to an increased number and higher proportion of 2^nd^ and 3^rd^ degree relatives, we had better resolution for familial effects in GS20K. Pedigree-associated genetics and nuclear family environment were now separable and the data structure in GS20K can provide sufficient evidence for both types of familial effects. For weight, urea, TC and HR, familial effects switched from nuclear family environment in GS10K to pedigree-associated genetics or pedigree-associated genetics plus nuclear family environment in GS20K. However, as in GS10K (Table 2 and S3 Table), there was still no evidence of either genetic or environmental familial effects for glucose and DBP in GS20K. The results from final selected models in GS20K are summarized in Fig 4.

The heritability estimate is nearly 90%, 60% and 60% for height, creatinine and HDL respectively, and for the remaining anthropometric and cardiometabolic traits, it ranges from 30%-50% and 20–30% for the two types of trait, respectively (Fig 4B). Although the proportion of genetic variance explained by SNP-associated and pedigree-associated genetic effects varies across traits, each genetic effect explains around 50% of the genetic variance on average (Fig 4C). In GS20K, the most commonly selected model was ‘**GKSC**’ (10 out of 16 times, Fig 4A and Table 3). SNP-associated genetic effects, pedigree-associated genetic effects, sibling environment and couple environment appeared in the final models for 16, 14, 12 and 16 out of 16 times respectively and the means of estimates for $h_{g}^{2},\;h_{kin}^{2},\;e_{s}^{2}$ and $e_{c}^{2}$ for traits which retained corresponding matrices (**GRM**_**g**_, **GRM**_**kin**_, **ERM**_**Sib**_ and **ERM**_**Couple**_ respectively) in the final models were of 0.20, 0.23, 0.05 and 0.11 respectively (Fig 4A and Table 3). For the nuclear family environment, the mean of estimates for $e_{f}^{2}$ for 4 traits which retained **ERM**_**Family**_ in final models was of 0.04 (Fig 4A and Table 3). On average across traits, our environmental matrices and the final selected models retained through our model selection procedure could explain ~16% and ~56% of the total phenotypic variance respectively (Fig 4B).

The major change in GS20K compared to GS10K is the significant evidence of effects of the sibling environment, particularly for cardiometabolic traits, resulting from the higher proportion of sibling relationships in GS20K (more than 12 times compared to GS10K, Table 1). However, the sibling effects were only 5% on average and were still relatively low compared to genetic effects and couple environment. Therefore, despite the change in population structure in GS20K, the major components for anthropometric and cardiometabolic traits were SNP-associated and pedigree-associated genetic effects and couple environment as they were in GS10K (Table 2).

## Discussion

The aim of this study was to better understand the architecture of human complex traits by dissecting phenotypic variation into SNP-associated additive genetic variation ($h_{g}^{2}$), pedigree-associated genetic variation ($h_{kin}^{2}$) and environmental influences of common environment shared by nuclear family members ($e_{f}^{2}$), full-siblings ($e_{s}^{2}$) and couples ($e_{c}^{2}$). We generated five design matrices **GRM**_**g**_, **GRM**_**kin**_, **ERM**_**Family**_, **ERM**_**Sib**_ and **ERM**_**Couple**_ to describe the five effects and we examined 16 human complex traits using genome-wide genotype data and genealogical information in the Generation Scotland: Scottish Family Health study (GS:SFHS) comprising samples from up to 20,000 individuals.

The results of these analyses suggest that SNP-associated genetic effects, pedigree-associated genetic effects and current environment shared by couples were the major contributors to phenotypic variation for anthropometric and cardiometabolic traits. Past environmental influences, such as shared sibling environment or nuclear family environment, made relatively small or undetectable contributions to trait variation (Table 2 and Table 3). The relative importance of a couple or spousal effect for most traits was also noted by Liu *et al*. [22], in analyses based only on pedigree relationships, although they did not find a significant spousal effect for cholesterol, HDL or glucose for which a significant couple effect was detected in this study.

Considering the low number of non-zero off-diagonal entries in **ERM**_**Couple**_ (1,283 or 1,767 pairs in GS10K or GS20K), the signal of couple effects was quite strong. We did observe significant phenotypic correlation between couple pairs for almost all traits in our data (S7 Table). For some traits this presumably represents current shared environment due to cohabitation, such as living habits and diet. For traits related to obesity, it is reasonable that current environmental effects are more important than past environmental effects since traits like BMI, fat, HDL and blood pressure are potentially influenced by recent food intake, exercise and medical treatment.

It should be noted that in our sample participants have an average age of ~50 years and individuals currently sharing a common household environment will largely be couples, whereas most individuals involved in sibling and parent-offspring relationships will no longer be cohabiting at the point when the data were recorded. It has been previously reported in obesity studies that common childhood environment only affects individuals in their mid-childhood but the influence does not last past adolescence [23,24]. Therefore, although the impacts of nuclear family or sibling environmental effects on the 16 traits we examined were relatively small, family and sibling environmental effects could be more important in younger cohorts and might be of greater importance for other complex traits and diseases where long-term environment may have an influence on a phenotype that is relatively stable over time.

For some traits, the most obvious example being height, couple effects may also, in part or completely, reflect assortative mating. A study by Keller *et al*. has shown that *h*^2^ estimate for height would be 13% higher with assortative mating than it would have been under random mating [23]. If there was assortative mating for any of the traits which retained **ERM**_**Couple**_ in final models but we modelled the couple correlation as an environmental effect, we would expect to obtain biased $e_{c}^{2}$ estimates. Moreover, modelling assortative mating as an environmental effect removes variance from the residual (“error”) variance. We therefore might obtain an inflated $h_{g}^{2}$ estimate if we have not taken assortative matting into account and reduce the residual variance as a consequence of modelling assortative matting as an environmental effect. In addition, assortative mating will have consequences for our interpretation of GWAS results as the combined effect of detected loci on the trait variance will be greater than the sum of the effects of the individual loci due to the positive correlations between loci. However, except for height, where the phenotype will be largely fixed by the time of marriage, for most traits it is difficult to determine whether assortative mating and/or shared environment are responsible for observed phenotypic correlations between couples.

Shared sibling environment was undetected for most of the traits in GS10K (Table 2), whereas there was significant evidence of it for many traits in GS20K (Table 3), indicating that the detection power of sibling environment benefits from the increase in number and proportion of sibling relationships (Table 1). Sibling effects, where detected, explained 5%, on average, of the trait variation. Estimated sibling effects may be inflated by non-additive genetics, (i.e. dominance and epistasis). As sibling effects only capture a fraction of the non-additive variation, the actual variation contributed by non-additive genetics might potentially be large and would merit further study.

Our analyses split the genetic variation approximately equally on average across traits between that which was associated with SNPs ($h_{g}^{2}$) and that which was associated with pedigree ($h_{kin}^{2}$). A plausible interpretation for the division of genetic effects into $h_{g}^{2}$ and $h_{kin}^{2}$ is that $h_{g}^{2}$ is able to explain the genetic variation attributed by common variants inherited from distant ancestors that are in LD at the population level and are well captured due to association with genotyped SNPs [12]. On the other hand, $h_{kin}^{2}$ accounts for the genetic variation due to rare variants, CNVs and other structural variation, etc. that cluster in specific families and are captured due to strong linkage in high-order pedigrees but are not in population-wide LD with common SNPs.

We compared $h_{g}^{2}$ and $h_{gkin}^{2}$ (calculated as $h_{g}^{2}+\;h_{kin}^{2}$) estimates obtained in final models from model selection in GS20K to two relevant publications from Zaitlen *et al*. [12] and Vattikuti *et al*. [19] that also explored the influence of including relatives on *h*^2^ estimation in family-based studies and compared $h_{gkin}^{2}$ estimates obtained in final models in GS20K to published twin studies [6,24–31]. Comparisons are shown in Table 4.

**Table 4:** Comparisons of the results from final models in GS20K to previous published results.

**Family-based GREML Studies**				
**Trait**	**Final models**		**Publications**	
	$h_{g}^{2}$**(*s*.*e*.)**	$h_{\mathrm{gkin}}^{2}$**(*s*.*e*.)**	$h_{g}^{2}$**(*s*.*e*.)**	$h_{\mathrm{gkin}}^{2}/h_{\mathrm{ped}}^{2}$^a^ **(*s*.*e*.)**
Height	0.43(0.02)	0.88(0.03)	0.40 [12]	0.69 [12]
BMI	0.25(0.02)	0.48(0.04)	0.14–0.23 [12,19]	0.34–0.42 [12,19]
WHR	0.11(0.02)	0.30(0.03)	0.06–0.13 [12,19]	0.19–0.28 [12,19]
Glucose	0.17(0.02)	0.17(0.02)	0.10 [19]	0.33 [19]
HDL	0.27(0.02)	0.56(0.03)	0.12–0.24 [12,19]	0.45–0.48 [12,19]
SBP	0.12(0.02)	0.19(0.03)	0.24 [19]	0.30 [19]
**Twin Studies**				
**Trait**	**Final models**		**Publications**	
	$h_{\mathrm{gkin}}^{2}$ **(*s*.*e*.)**		$h_{\mathrm{ped}}^{2}$ **(*s*.*e*.)**	
Height	0.88(0.03)		0.89–0.93 [6]	
Weight	0.54(0.04)		0.64–0.84 [24]	
Fat	0.49(0.04)		0.59–0.63 [25]	
BMI	0.48(0.04)		0.48–0.61 [26]	
Hips	0.42(0.04)		0.52–0.58 [25]	
Waist	0.50(0.03)		0.46 [27]	
WHR	0.30(0.03)		0.31 [27]	
Urea	0.25(0.03)		0.36–0.54 [29]	
Creatinine	0.60(0.03)		0.37 [28]	
Glucose	0.17(0.02)		0.45 [30]	
TC	0.33(0.03)		0.46–0.57 [26]	
HDL	0.56(0.03)		0.50–0.62 [26]	
SBP	0.19(0.03)		0.57 [31]	
DBP	0.16(0.02)		0.45 [31]	
HR	0.26(0.03)		0.64 [31]	

^a^ $h_{gkin}^{2}$ is an equivalent estimate to $h_{ped}^{2}$ but is calculated using genomic information

When comparing with two family-based GREML studies (Table 4), our $h_{g}^{2}$ and $h_{gkin}^{2}$ estimates from final models are generally higher than published relevant results, except for the $h_{g}^{2}$ estimate for SBP and the $h_{gkin}^{2}$ estimates for glucose and SBP. When comparing with twin studies (Table 4), our $h_{gkin}^{2}$ estimates for all anthropometric traits, urea, TC and HDL given by final selected models in GS20K are reasonably close to reported $h_{ped}^{2}$ estimates, which suggests little missing heritability. Hence, our results provide no evidence that heritabilities given by previous twin studies were inflated for these traits. For glucose, SBP, DBP and HR, however, our $h_{gkin}^{2}$ estimates are significantly lower than previously published estimates of $h_{ped}^{2}$, whereas for creatinine, $h_{gkin}^{2}$ is significantly larger.

To validate the analytical approach used in this study and to evaluate model robustness, we conducted a detailed simulation study using real genotype and pedigree information obtained from GS10K. The simulation results confirmed that our models were generally robust (S5 Table). However, the inevitable correlations between our design matrices can, under some circumstances, make it challenging to partition variance for correlated factors in variance component analyses and accurately discriminate between competing models in model selection. Nonetheless, any influence of inaccurately partitioning variance among correlated matrices was relatively limited and our models were always able to provide us with a good idea of the magnitude of corresponding effects as the mean estimate for each parameter was always very close the simulated settings when the model used for analysis matched the simulated sources of trait variation.

The effectiveness of the model selection procedure was also validated using the simulated data with the model selection procedure often (≥80%) resulting in models containing all major phenotype components (S6 Table). However, due to the limited number of appropriate relationships in GS10K to resolve correlations between matrices and to detect factors with small effects, our model selection procedure may omit minor effects (contributing 5% or less of the trait variance, for example). In addition, the procedure may sometimes identify incorrect models (not being able to distinguish familial effects as mentioned in the simulation study and S6 Table) and this might be the case for weight, urea, TC and HR in Table 2. However, with sufficient data from higher order pedigree relationships, as was the case in GS20K, the impact of covariances between design matrices in first order relatives (parent-offspring, siblings and couples) are mitigated and further components of variance became separable (Table 3).

To sum up, we provide evidence that for the traits we have analysed, heritabilities are divided approximately evenly between pedigree-associated and SNP-associated genetic effects. This is the case even when, as here, we have taken care to consider various models of environmental covariation of first-degree relatives (including couples). It appears that confounding factors like dominance, shared full-sibling environment and the past rearing environment seem to have relatively small contribution to phenotypic variation for these traits in our population. We find that current shared environment of couples is able to account for another ~11% on average of the phenotypic variation of human complex traits. This has been seldom mentioned in previous heritability studies but we note that as an effect that inflates the covariance between nominally unrelated individuals, it should not substantially bias or inflate $h_{ped}^{2}$ and $h_{gkin}^{2}$. It should be taken into account that couple effects may also be present in cohorts of unrelated individuals which may often include couples but ignore any correlation between them. Therefore, it might bias $h_{g}^{2}$ from genotype-based studies which do not account for such couple effects and could have an impact on GWAS studies.

Overall, our work shows that SNP-associated genetic effects, pedigree-associated genetic effects and current shared couple environmental effects are three fundamental components of phenotypic variation for traits related to anthropometrics and cardiometabolism and current shared environmental effects have more impact than past shared environmental effects. This also has implications for models to be used in further studies of the architecture of complex traits including utilising the appropriate models for GWAS and related analyses and for personalised disease risk prediction.

## Materials and Methods

### Ethics statement

The data were obtained from the Generation Scotland: Scottish Family Health Study (GS:SFHS). Ethical approval for the study was given by the NHS Tayside committee on research ethics (reference 05/s1401/89) and participants provided written consent. Governance of the study, including public engagement, protocol development and access arrangements, was overseen by an independent advisory board, established by the Scottish government

### Data description

Our dataset came from the Generation Scotland Scottish Family Health Study (GS:SFHS) project (http://www.generationscotland.org), which was collected by a cross-disciplinary collaboration of Scottish medical schools and the National Health Service (NHS) from Feb 2006 to Mar 2011 [21,32].

Data for 16 complex traits were used. These were 8 anthropometric traits: height, weight, fat, body mass index ($\text{BMI}=\frac{\text{Weight}}{{\text{Height}}^{2}}$), hips, waist, waist-to-hips ratio (WHR) and a body shape index (ABSI $=\frac{\text{Waist Circumference}\times {\text{Height}}^{5/6}}{{\text{Weight}}^{2/3}}$) [20] and 8 cardiometabolic traits: levels of creatinine, urea, total cholesterol (TC) and high density lipoprotein (HDL) in serum and glucose in blood after a four hour fast period, systolic blood pressure (SBP), diastolic blood pressure (DBP) and heart rate (HR). None of the traits was adjusted for medication or fasting status. We explored the phenotypic distributions of these traits and conducted natural logarithm transformations for them, except for height, sodium and fat, to obtain approximate normal distributions. We set phenotypes with values greater or smaller than the mean ± 4 standard deviations (after adjusting for sex, age and age^2^) to missing.

Data also contained the information of sex, age, clinics where the phenotypes were measured and Scottish Index of Multiple Deprivation (SIMD, an environmental ranking based on living areas, [33]). A descriptive analysis can be seen in S1 Table.

The first set of analyses presented in the manuscript are based on a data set of nearly 10,000 individuals from GS:SFHS (GS10K). These have multiple degrees of kinships, including 5,061 family members from 1,612 nuclear or extended families, and were genotyped with the Illumina OMNiExpress chip (707,686 SNPs). We conducted data quality control in Plink v1.07 [34] and GenABEL v1.7–6 [35]. SNPs with a minor allele frequency (MAF) < 0.05, a Hardy-Weinberg Equilibrium’s (HWE) p-value <10^−6^ and a missingness > 2% were excluded. Duplicate samples, gender discrepancies and individuals with more than 5% missingness were also removed. After the quality control we kept 9,863 individuals genotyped for 550,796 common SNPs over the 22 autosomes.

An extended dataset (GS20K) was used to validate the results obtained with GS10K and evaluate the effect of including further close relationships in our data. The extra 10,000 individuals were genotyped with the same chip and quality control was performed using the same criteria as in the GS10K. After quality control, GS20K consisted of 20,032 individuals, 18,293 of whom came from 6,578 nuclear or extended families, and 519,729 common SNPs across the 22 autosomes.

A comparison of the difference in relationships between GS10K and GS20K can be seen in Table 1.

### Statistical methods

Our model allows trait variation to be influenced by the genetic effects associated with SNPs ($h_{g}^{2}$) and pedigree ($h_{kin}^{2}$) and the environmental effects shared by families ($e_{f}^{2}$), couples ($e_{c}^{2}$) and full-siblings ($e_{s}^{2}$), (Fig 1). To estimate the influence of each effect, we generated five design matrices: **GRM**_**g**_, **GRM**_**kin**_, **ERM**_**Family**_, **ERM**_**Sib**_ and **ERM**_**Couple**_.

### Genomic relationship matrices

A genomic relationship matrix (GRM) contains estimated genomic relatedness between pairs of individuals calculated from identity-by-state marker relationships as in Yang *et al*. [16,17].

Each off-diagonal entry in the GRM represents the realised genomic relationship between a pair of individuals:

$$\frac{1}{N}\sum_{i=1}^{N}\frac{\left(x_{ji}-2p_{i})(x_{ki}-2p_{i}\right)}{2p_{i}(1-p_{i})}$$

where, *p*_*i*_ is the minor allele frequency (MAF) for SNP *i*, *x*_*ji*_ or *x*_*ki*_ is the allelic dose for individual *j* or *k* at locus *i* (*x* = 2 if the individual carries two rare alleles, *x* = 1 if the individual is heterozygous, *x* = 0 if the individual carries two common alleles) and *N* is the total number of SNPs.

Each entry on the diagonal represents the inbreeding coefficient calculated as:

$$1+\frac{1}{N}\sum_{i=1}^{N}\frac{x_{ji}^{2}-(1+2p_{i})x_{ji}+{2p}_{i}^{2}}{2p_{i}(1-p_{i})}$$

We used GCTA [16] to generate **GRM**_**g**_ and obtained **GRM**_**kin**_ by modification of **GRM**_**g**_ in R [36]. Their definitions are identical to matrices **K**_**IBS**_ and **K**_**IBS**>*t*_ in Zaitlen *et al*. [12] respectively.

**GRM**_**g**_: a GRM estimated using all common SNPs, and designed to capture the additive genetic variance explained by common SNPs in the population sample.

**GRM**_**kin**_: a modified GRM calculated as in Zaitlen *et al*. [12] designed to estimate the extra genetic effects associated with pedigree, the variance explained by shared genetic factors in close relatives. **GRM**_**kin**_ was created by setting to 0 all entries in **GRM**_**g**_ smaller than 0.025.

The number of entries different from 0 in each of the matrices is shown in Table 1.

### Environmental relationship matrices

An environmental relationship matrix (ERM) is a covariance matrix designed to capture the variance due to common environmental effects shared among a specified group of individuals.

The ERM coefficient for each pair of individuals is 1 in if they share a particular environment, e.g., living in the same area or coming from the same family; otherwise, it is 0. Each entry on the diagonal is 1.

We generated 3 different ERMs in R [36]: **ERM**_**Couple**_, **ERM**_**Sib**_ and **ERM**_**Family**_.

**ERM**_**Couple**_: **ERM**_**Couple**_ was designed to capture the common environmental effects shared between a couple. The ERM coefficient of two individuals was 1 if they were identified as a couple, defined as a pair of individuals with at least one offspring within GS:SFHS. Each entry on the diagonal was 1.

**ERM**_**Sib**_: **ERM**_**Sib**_ was designed to capture the common environmental effects shared between full-siblings. The ERM coefficient of two individuals was 1 if they were identified as full-siblings. Each diagonal entry was 1.

**ERM**_**Family**_: **ERM**_**Family**_ was designed to capture the common environmental effects shared within each nuclear family comprising parents and offspring. The ERM coefficient of two individuals was 1 if they were identified as a parent-offspring pair, full-siblings or a couple. The ERM coefficient of two individuals was 1 if they were identified as nuclear family members, including parent-offspring, couple and full-sibling relationships. Each diagonal entry was 1.

The number of entries different from 0 in each of the environmental matrices is shown in Table 1. Details about model and matrices we defined can be seen in Fig 1.

### Variance component analysis

We used the genomic and environmental matrices described above to partition the phenotypic variance observed for the traits using a mixed model in a restricted maximum likelihood (REML) framework. The analyses were implemented in GCTA [16]. The equations used to evaluate each model were the subsets of the full model:

$$y=X\beta +g_{g}+g_{\mathrm{kin}}+e_{f}+e_{s}+e_{c}+\varepsilon \;,\;\text{with}$$

$$V={\mathrm{GRM}}_{g}\sigma_{g}^{2}+{\mathrm{GRM}}_{\mathrm{kin}}\sigma_{kin}^{2}+{\mathrm{ERM}}_{\mathrm{Family}}\sigma_{ef}^{2}+{\mathrm{ERM}}_{\mathrm{Sib}}\sigma_{es}^{2}+{\mathrm{ERM}}_{\mathrm{Couple}}\sigma_{ec}^{2}+I\sigma_{\varepsilon }^{2}$$

where **y** is an *n* × 1 vector of observed phenotypes with *n* being the sample size (number of individuals), and **V** the total phenotypic variance matrix, **β** is an *m* × 1 vector of fixed effects with *m* being the total level of covariates and **X** its design matrix with dimension *n* × *m*, **g**_**g**_ is an *n* × 1 vector of the total additive genetic effects of the individuals captured by genotyped SNPs with $g_{g}∼N\left(0,{\mathrm{GRM}}_{g}\sigma_{g}^{2}\right)$, **g**_**kin**_ is an *n* × 1 vector of the extra genetic effects associated with the pedigree for relatives with $g_{\mathrm{kin}}∼N\left(0,{\mathrm{GRM}}_{\mathrm{kin}}\;\sigma_{kin}^{2}\right)$, **e**_**f**_, **e**_**s**_ and **e**_**c**_ are *n* × 1 vectors representing the common environmental effects shared by nuclear family members, full-siblings and couples with $e_{f}∼N(0,{\mathrm{ERM}}_{\mathrm{Family}}\;\sigma_{ef}^{2})$, $e_{s}∼N(0,{\mathrm{ERM}}_{\mathrm{Sib}}\;\sigma_{es}^{2})$ and $e_{c}∼N(0,{\mathrm{ERM}}_{\mathrm{Couple}}\;\sigma_{ec}^{2})$ and **ε** is an *n* × 1 vector of residuals. We fitted a range of models including different combinations of effects, and named them using abbreviations according to the effects used. We used the codes ‘**G**’ for **GRM**_**g**_, ‘**K**’ for **GRM**_**kin**_, ‘**F**’ for **ERM**_**Family**_, ‘**S**’ for **ERM**_**Sib**_ and ‘**C**’ for **ERM**_**Couple**_ –e.g. ‘**GKC**’ = **GRM**_**g**_ + **GRM**_**kin**_ + **ERM**_**Couple**_, and the proportion of total phenotypic variance captured by each matrix was termed $h_{g}^{2},\;h_{kin}^{2},\;e_{f}^{2},\;e_{s}^{2}$ and $e_{c}^{2}$ accordingly. All models include a residual matrix and the total heritability $h_{gkin}^{2}$ is always the sum of $h_{g}^{2}\;+\;h_{kin}^{2}$ for any model.

There were 31 different models from all the possible combinations of the five matrices. The abbreviations for each model and the formulae to estimate each term in each model are listed in S2 Table. The results for each model are listed in S4 Table.

In addition to the matrices described (including the residual matrix), we always included the fixed effects of sex, age, age^2^, sex-by-age interaction, clinic, standardised SIMD and SIMD^2^ and the first 20 eigenvectors of **GRM**_**g**_ (to ameliorate problems associated with data structure).

### Stepwise model selection

We conducted a stepwise model selection to find the most appropriate genetic and environmental model for each trait and dissect the phenotypic variation into its components (SNP-associated additive genetic variance, pedigree-associated genetic effects shared among relatives and common environmental effects shared among the specified groups including nuclear family members, couples and full-siblings).

The stepwise selection began with the full model ‘**GKFSC**’, where all matrices were fitted together. We performed a Wald test and a log-likelihood ratio test (LRT, using a mixture distribution of $\chi_{df=0}^{2}$ and $\chi_{df=1}^{2}$ with a probability of 0.5 [16]) for each component and removed the component, if any, that was non-significant for both tests at α = 5% level and had the highest p-value for the Wald test. We repeated this process until all the remaining components were significant for at least one test. We did not correct for the limited number of traits analysed so error rates in this procedure should be considered to be on a per trait basis.

### Simulation study

In order to evaluate the robustness of our models and the performance of our stepwise model selection, we conducted a simulation study. We simulated, based on the real genotypic information and the real pedigree, different sets of phenotypes for each of the 9,863 individuals in GS10K.

For simulating the genetic effects, we used a similar approach to Zaitlen *et al*. [12] by dividing the genome into two: even and odd chromosomes, and randomly selecting 550 SNPs from even and odd chromosomes (approximately 1 from each 500 SNPs), representing the observed causal loci that were in LD with the SNPs (SNP-associated genetic effects) and the unobserved genetic variants that were not in LD with the SNP array (pedigree-associated genetic effects) separately. In a later step, only even chromosomes were used to generate **GRM**_**g**_ and **GRM**_**kin**_. Each locus was assigned an effect size driven from exponential distribution as in Fisher [37] and the summed effects for even and odd chromosome SNPs were designed to explain $h_{g}^{2}$ and $h_{kin}^{2}$ of the trait variance respectively.

For environmental factors, we simulated a sibling environmental effect, a couple environmental effect and two nuclear family environmental effects (youth and adulthood environments) for each individual. The corresponding effect sizes for sibling, couple and nuclear family environmental effects were derived from $N(0,\;e_{s}^{2})$, $N(0,\;e_{c}^{2})$ and $N(0,\;e_{f}^{2})$ accordingly and were the same among full-siblings, between couples and among nuclear family members.

In addition, we simulated a random residual effect for each individual, the residuals were derived from $N(0,\;e_{e}^{2})$ where $e_{e}^{2}$ represents the proportion of variance remaining in each of the scenarios. For each scenario, each component ($h_{g}^{2},h_{kin}^{2},e_{c}^{2},e_{s}^{2},e_{f}^{2}$) was given a proportion of the variance explained and $e_{e}^{2}$ was $1-h_{g}^{2}-h_{kin}^{2}-e_{c}^{2}-e_{s}^{2}-e_{f}^{2}$. The final phenotypes would be the sum of these genetic and environmental effects and residuals, and the expected mean and variance of simulated phenotypes were 0 and 1, respectively. More details about how we simulated phenotypes can be found in S1 Text.

We evaluated the robustness of our models under situations where phenotypes were contributed by i) one of the five effects, ii) SNP-associated genetic effects and one of the familial effects (either pedigree-associated genetic effects or nuclear family environmental effects) and iii) SNP-associated genetic effects, familial effects and other environmental effects. All scenarios included residuals and 50 to 100 replicates were analysed for each scenario. The results of simulations were evaluated using a Z-test, which tested whether the mean estimate for each parameter deviated significantly from its simulated value. Note, it was too time consuming to explore all the possible combinations of models and simulated phenotypes, therefore, we mainly focused on the models that were selected in model selection procedure for the real phenotypes in GS10K (Table 2) as well as the fundamental models of our study. More details about the parameter settings for these scenarios can be found in S5 Table.

**ERM**_**Family**_ posited a relationship between siblings, parents-offspring and couples is somewhat confounded with the addition of **GRM**_**kin**_ and **ERM**_**Couple**_, making separation and estimation of these effects ($e_{f}^{2}$, $h_{kin}^{2}$ and $e_{c}^{2}$) challenging, as confirmed by the results from analysis of real phenotypes in GS10K (Table 2). Hence, we evaluated the effectiveness of our model selection procedure under situations where phenotypes were contributed by moderate SNP-associated genetic effects and low sibling environmental effects plus a) moderate nuclear family environmental effects but low pedigree-associated genetic effects and couple environmental effects, b) low nuclear family environmental effects but moderate pedigree-associated genetic effects and couple environmental effects and c) moderate nuclear family environmental effects, pedigree-associated genetic effects and couple environmental effects. All scenarios included residuals. More details about the parameter settings for these scenarios can be found in S6 Table. We conducted the model selection procedure for each replicate to see whether the final model selected matched the simulated phenotypic components for these scenarios (Note: we ran 10 replicates for each scenario here). In addition, variance component analyses were performed using final selected models for these replicates to see whether the estimates of parameters were close to their simulated values.

## Supporting Information

S1 Text Simulating phenotypes. (DOCX)

S1 Table Descriptive analysis of traits and covariates using GS10K data. (XLSX)

S2 Table Abbreviations and equations for terms for all 31 possible alternative models used in our study. (DOCX)

S3 Table Heritability estimates using model 'G' in subpopulations of GS10K with different GRM cut-offs. (XLSX)

S4 Table Results of variance component analysis using alternative model for 16 traits in GS10K. (XLSX)

S5 Table Parameter settings and main results (mean and 95% CI) for all scenarios in simulation study using GS10K data. (XLSX)

S6 Table Parameter settings for phenotypes simulated using GS10K data, results of model selection using simulated phenotypes and results of variance component analyses using selected models. (XLSX)

S7 Table Phenotypic correlation for spousal pairs (covariates adjusted) in GS10K. (XLSX)
