# Correction: Pedigree- and SNP-Associated Genetics and Recent Environment are the Major Contributors to Anthropometric and Cardiometabolic Trait Variation

**DOI:** 10.1371/journal.pgen.1006608

**Published:** 2017-02-14

**Authors:** Charley Xia, Carmen Amador, Jennifer Huffman, Holly Trochet, Archie Campbell, David Porteous, Generation Scotland, Nicholas D. Hastie, Caroline Hayward, Veronique Vitart, Pau Navarro, Chris S. Haley

The following information is missing from the Funding section: Genotyping of the GS:SFHS samples was carried out by the Genetics Core Laboratory at the Wellcome Trust Clinical Research Facility, Edinburgh, Scotland and was funded by the Medical Research Council UK and the Wellcome Trust (Wellcome Trust Strategic Award “STratifying Resilience and Depression Longitudinally” (STRADL) Reference 104036/Z/14/Z).

These additional funders had no role in study design, data collection and analysis, decision to publish, or preparation of the manuscript.
