# Supplementary material for: Pedigree- and SNP-Associated Genetics and Recent Environment are the Major Contributors to Anthropometric and Cardiometabolic Trait Variation
Source: PLoS Genet. 2016 Feb 2;12(2):e1005804. doi: 10.1371/journal.pgen.1005804 (PMC4737500; doi:10.1371/journal.pgen.1005804)
Supplement: S1 Text — (DOCX) [file pgen.1005804.s001.docx]

**S1 Text: Simulating phenotypes**

In order to evaluate the robustness of our models and the performance of our stepwise model selection, we conducted a simulation study. We simulated, based on the real genotypic information and the real pedigree, different sets of phenotypes for each of the 9,863 individuals in GS10K. The simulated phenotypes were generated by combining various proportions of simulated effects for SNP-associated genetics, pedigree-associated genetics, nuclear family environment, shared couple environment and sibling environment.

**Simulating phenotypes using real genotypes and pedigree**

For simulating the genetic effects we used a similar approach to Zaitlen *et al.* [[1](#_ENREF_1)], but based on our real genotype information in GS10K. The genome was divided into two: even chromosomes were used to create the observed genetic effects that were in LD with the SNPs (in a later step, only even chromosomes will be used to generate the appropriate genomic relationship matrices); odd chromosomes were used to create the unobserved variants that were not in LD with the SNP array. We randomly selected 1 in every 500 SNPs (MAF > 0.05) on even chromosomes, ending up with 550 ‘causal loci’ representing the causal variants tagged by genotyping platform. We assigned an effect to the rare alleles of the selected markers (assuming an additive model). The summed effect for those loci was $\mathbf{g}_{\mathbf{g}}$ and was calculated as $\sum_{i=1}^{N} a_{i}x_{i}$ for each individual, where *N* is the number of causal loci, $a_{i}$ is the effect size of allele *i* and $x_{i}$ is the allelic dose for allele *i*. Similarly, another 550 common ‘causal loci’ were randomly selected on odd chromosomes, representing the variants that were not in LD with the SNP array. The summed effect for those loci was $\mathbf{g}_{\mathbf{kin}}$, which was calculated using the same formula as $\mathbf{g}_{\mathbf{g}}$.

These basic genetic settings were the same as in Zaitlen *et al.* [[1](#_ENREF_1)], except for the assumption we used for effect sizes. The effect sizes in our simulation study were derived from an exponential distribution as in Fisher [[2](#_ENREF_2)]. The distributions of effect sizes for ‘casual loci’ on even and odd chromosomes were $\boldsymbol{a}_{\boldsymbol{g}}\sim E(\lambda=\sqrt{\frac{4\times N\times\bar{pq}}{h_{g}^{2}}}$) and $\boldsymbol{a}_{\boldsymbol{kin}}\sim E(\lambda=\sqrt{\frac{4\times N\times\bar{pq}}{h_{kin}^{2}}}$) respectively, where $\boldsymbol{a}_{\boldsymbol{g}}$ and $\boldsymbol{a}_{\boldsymbol{kin}}$ are *N* × 1 vectors of effect sizes for chosen SNPs on even and odd chromosomes separately and $\bar{pq}$ is the mean of minor allele frequency times major allele frequency for these loci, which is 0.1825 here.

We transformed $\mathbf{g}_{\mathbf{g}}$ and $\mathbf{g}_{\mathbf{kin}}$ to normal distributions with mean equal to 0 and variance equal to $h_{g}^{2}$ or $h_{kin}^{2}$, which are the proportion of the variance of the simulated phenotypes explained by variants in LD with the markers, and variants not in LD with the markers, respectively.

The environmental effects were simulated based on the real pedigree. For sibling environment ($\mathbf{e}_{\mathbf{s}}$) and couple environment ($\mathbf{e}_{\mathbf{c}}$), the effect sizes were derived from two normal distributions: $N(0, e_{s}^{2})$ and $N(0, e_{c}^{2})$ respectively. We assigned the same random couple effect to each pair of individuals in a couple and the same random sibling effect to each of the full-siblings from the same nuclear family. Individuals without any spouse or/and siblings in the data were also given a random couple effect and a random sibling effect that was unique to themselves. For the nuclear family environment ($\mathbf{e}_{\mathbf{f}}$), individuals were given two nuclear family effects: one for their youth (representing familial environment when living with their parents) and the other for adulthood (familial environment when living alone or with their spouse and children). Nuclear family members shared the same nuclear family effect, whereas single individuals did not share nuclear family effect with any other individuals. Therefore, individuals with parents and with a spouse or/and offspring (1,305 individuals in GS10K) shared two separate familial effects, one with their parents and any sibs and one with their spouse and/or children; individuals without any first degree relatives (1,785 individuals in GS10K) had two unique familial effects; and the remaining individuals (6,773 individuals in GS10K) had one shared and one unique familial effects. Both family environments (youth and adulthood) contributed equally to create the final family environmental effect. The rationale for this approach is that in three generation families (grandparents, parents and progeny) it allows separate nuclear family environment effects for the grandparents and their grandprogeny, i.e. it does not assume that they share the same family environment. The effect size was randomly drawn from $N(0, e_{f}^{2})$.

As before, we transformed $\mathbf{e}_{\mathbf{f}}$, $\mathbf{e}_{\mathbf{s}}$ and $\mathbf{e}_{\mathbf{c}}$ to normal distributions with mean equal to 0 and variance equal to $e_{f}^{2}$, $e_{s}^{2}$ or $e_{c}^{2}$, being $e_{f}^{2}$, $e_{s}^{2}$ or $e_{c}^{2}$ the proportion of the variance of the simulated phenotype explained by family, sibling or couple environment, respectively.

We also simulated a random residual effect for each individual ($\boldsymbol{\varepsilon}$), the residuals were derived from $N(0,e_{e}^{2})$ where $e_{e}^{2}$ represents the proportion of variance remaining in each of the scenarios. For each scenario, each component ($h_{g}^{2},h_{kin}^{2},e_{c}^{2},e_{s}^{2},e_{f}^{2}$) was given a proportion of the variance explained (0% - 50%) and $e_{e}^{2}$ was $1-h_{g}^{2}-h_{kin}^{2}-e_{c}^{2}-e_{s}^{2}-e_{f}^{2}$. The final phenotypes would be the sum of transformed$\mathbf{g}_{\mathbf{g}}$, $\mathbf{g}_{\mathbf{kin}}$, $\mathbf{e}_{\mathbf{f}}$**,** $\mathbf{e}_{\mathbf{s}}$**,** $\mathbf{e}_{\mathbf{c}}$ and $\boldsymbol{\varepsilon}$**,** and the expected mean and variance of simulated phenotypes was 0 and 1, respectively.

**Reference**

1. Zaitlen N, Kraft P, Patterson N, Pasaniuc B, Bhatia G, et al. (2013) Using extended genealogy to estimate components of heritability for 23 quantitative and dichotomous traits. PLoS Genet 9: e1003520.

2. Fisher RA (1930) The Genetical Theory of Natural Selection. Oxford: Clarendon.
