# Supplementary material for: Pedigree- and SNP-Associated Genetics and Recent Environment are the Major Contributors to Anthropometric and Cardiometabolic Trait Variation
Source: PLoS Genet. 2016 Feb 2;12(2):e1005804. doi: 10.1371/journal.pgen.1005804 (PMC4737500; doi:10.1371/journal.pgen.1005804)
Supplement: S2 Table — (DOCX) [file pgen.1005804.s003.docx]

| **S2 Table.** Abbreviations and equations for terms for all 31 possible alternative models used in our study | | | | | |
| --- | --- | --- | --- | --- | --- |
| **Model** | **GRM_g_** | **GRM_kin_** | **ERM_Family_** | **ERM_Sib_** | **ERM_Couple_** |
| **G** | $\boldsymbol{h}_{\boldsymbol{g}}^{\boldsymbol{2}}\boldsymbol{=}\frac{\boldsymbol{\sigma}_{\boldsymbol{g}}^{\boldsymbol{2}}}{\boldsymbol{\sigma}_{\boldsymbol{g}}^{\boldsymbol{2}}\boldsymbol{+}\boldsymbol{\sigma}_{\boldsymbol{\varepsilon}}^{\boldsymbol{2}}}$ |  |  |  |  |
| **K** |  | $\boldsymbol{h}_{\boldsymbol{kin}}^{\boldsymbol{2}}\boldsymbol{=}\frac{\boldsymbol{\sigma}_{\boldsymbol{kin}}^{\boldsymbol{2}}}{\boldsymbol{\sigma}_{\boldsymbol{kin}}^{\boldsymbol{2}}\boldsymbol{+}\boldsymbol{\sigma}_{\boldsymbol{\varepsilon}}^{\boldsymbol{2}}}$ |  |  |  |
| **F** |  |  | $\boldsymbol{e}_{\boldsymbol{f}}^{\boldsymbol{2}}\boldsymbol{=}\frac{\boldsymbol{\sigma}_{\boldsymbol{ef}}^{\boldsymbol{2}}}{\boldsymbol{\sigma}_{\boldsymbol{ef}}^{\boldsymbol{2}}\boldsymbol{+}\boldsymbol{\sigma}_{\boldsymbol{\varepsilon}}^{\boldsymbol{2}}}$ |  |  |
| **S** |  |  |  | $\boldsymbol{e}_{\boldsymbol{s}}^{\boldsymbol{2}}\boldsymbol{=}\frac{\boldsymbol{\sigma}_{\boldsymbol{es}}^{\boldsymbol{2}}}{\boldsymbol{\sigma}_{\boldsymbol{es}}^{\boldsymbol{2}}\boldsymbol{+}\boldsymbol{\sigma}_{\boldsymbol{\varepsilon}}^{\boldsymbol{2}}}$ |  |
| **C** |  |  |  |  | $\boldsymbol{e}_{\boldsymbol{c}}^{\boldsymbol{2}}\boldsymbol{=}\frac{\boldsymbol{\sigma}_{\boldsymbol{ec}}^{\boldsymbol{2}}}{\boldsymbol{\sigma}_{\boldsymbol{ec}}^{\boldsymbol{2}}\boldsymbol{+}\boldsymbol{\sigma}_{\boldsymbol{\varepsilon}}^{\boldsymbol{2}}}$ |
| **GK** | $\boldsymbol{h}_{\boldsymbol{g}}^{\boldsymbol{2}}\boldsymbol{=}\frac{\boldsymbol{\sigma}_{\boldsymbol{g}}^{\boldsymbol{2}}}{\boldsymbol{\sigma}_{\boldsymbol{g}}^{\boldsymbol{2}}\boldsymbol{+}\boldsymbol{\sigma}_{\boldsymbol{kin}}^{\boldsymbol{2}}\boldsymbol{+}\boldsymbol{\sigma}_{\boldsymbol{\varepsilon}}^{\boldsymbol{2}}}$ | $\boldsymbol{h}_{\boldsymbol{kin}}^{\boldsymbol{2}}\boldsymbol{=}\frac{\boldsymbol{\sigma}_{\boldsymbol{kin}}^{\boldsymbol{2}}}{{\boldsymbol{\sigma}_{\boldsymbol{g}}^{\boldsymbol{2}}\boldsymbol{+\sigma}}_{\boldsymbol{kin}}^{\boldsymbol{2}}\boldsymbol{+}\boldsymbol{\sigma}_{\boldsymbol{\varepsilon}}^{\boldsymbol{2}}}$ |  |  |  |
| **GF** | $\boldsymbol{h}_{\boldsymbol{g}}^{\boldsymbol{2}}\boldsymbol{=}\frac{\boldsymbol{\sigma}_{\boldsymbol{g}}^{\boldsymbol{2}}}{\boldsymbol{\sigma}_{\boldsymbol{g}}^{\boldsymbol{2}}\boldsymbol{+}\boldsymbol{\sigma}_{\boldsymbol{ef}}^{\boldsymbol{2}}\boldsymbol{+}\boldsymbol{\sigma}_{\boldsymbol{\varepsilon}}^{\boldsymbol{2}}}$ |  | $\boldsymbol{e}_{\boldsymbol{f}}^{\boldsymbol{2}}\boldsymbol{=}\frac{\boldsymbol{\sigma}_{\boldsymbol{ef}}^{\boldsymbol{2}}}{\boldsymbol{\sigma}_{\boldsymbol{g}}^{\boldsymbol{2}}\boldsymbol{+}\boldsymbol{\sigma}_{\boldsymbol{ef}}^{\boldsymbol{2}}\boldsymbol{+}\boldsymbol{\sigma}_{\boldsymbol{\varepsilon}}^{\boldsymbol{2}}}$ |  |  |
| **GS** | $\boldsymbol{h}_{\boldsymbol{g}}^{\boldsymbol{2}}\boldsymbol{=}\frac{\boldsymbol{\sigma}_{\boldsymbol{g}}^{\boldsymbol{2}}}{\boldsymbol{\sigma}_{\boldsymbol{g}}^{\boldsymbol{2}}\boldsymbol{+}\boldsymbol{\sigma}_{\boldsymbol{es}}^{\boldsymbol{2}}\boldsymbol{+}\boldsymbol{\sigma}_{\boldsymbol{\varepsilon}}^{\boldsymbol{2}}}$ |  |  | $\boldsymbol{e}_{\boldsymbol{s}}^{\boldsymbol{2}}\boldsymbol{=}\frac{\boldsymbol{\sigma}_{\boldsymbol{es}}^{\boldsymbol{2}}}{\boldsymbol{\sigma}_{\boldsymbol{g}}^{\boldsymbol{2}}\boldsymbol{+}\boldsymbol{\sigma}_{\boldsymbol{es}}^{\boldsymbol{2}}\boldsymbol{+}\boldsymbol{\sigma}_{\boldsymbol{\varepsilon}}^{\boldsymbol{2}}}$ |  |
| **GC** | $\boldsymbol{h}_{\boldsymbol{g}}^{\boldsymbol{2}}\boldsymbol{=}\frac{\boldsymbol{\sigma}_{\boldsymbol{g}}^{\boldsymbol{2}}}{\boldsymbol{\sigma}_{\boldsymbol{g}}^{\boldsymbol{2}}\boldsymbol{+}\boldsymbol{\sigma}_{\boldsymbol{ec}}^{\boldsymbol{2}}\boldsymbol{+}\boldsymbol{\sigma}_{\boldsymbol{\varepsilon}}^{\boldsymbol{2}}}$ |  |  |  | $\boldsymbol{e}_{\boldsymbol{c}}^{\boldsymbol{2}}\boldsymbol{=}\frac{\boldsymbol{\sigma}_{\boldsymbol{ec}}^{\boldsymbol{2}}}{\boldsymbol{\sigma}_{\boldsymbol{g}}^{\boldsymbol{2}}\boldsymbol{+}\boldsymbol{\sigma}_{\boldsymbol{ec}}^{\boldsymbol{2}}\boldsymbol{+}\boldsymbol{\sigma}_{\boldsymbol{\varepsilon}}^{\boldsymbol{2}}}$ |
| **KF** |  | $\boldsymbol{h}_{\boldsymbol{kin}}^{\boldsymbol{2}}\boldsymbol{=}\frac{\boldsymbol{\sigma}_{\boldsymbol{kin}}^{\boldsymbol{2}}}{\boldsymbol{\sigma}_{\boldsymbol{kin}}^{\boldsymbol{2}}\boldsymbol{+}\boldsymbol{\sigma}_{\boldsymbol{ef}}^{\boldsymbol{2}}\boldsymbol{+}\boldsymbol{\sigma}_{\boldsymbol{\varepsilon}}^{\boldsymbol{2}}}$ | $\boldsymbol{e}_{\boldsymbol{f}}^{\boldsymbol{2}}\boldsymbol{=}\frac{\boldsymbol{\sigma}_{\boldsymbol{ef}}^{\boldsymbol{2}}}{\boldsymbol{\sigma}_{\boldsymbol{kin}}^{\boldsymbol{2}}\boldsymbol{+}\boldsymbol{\sigma}_{\boldsymbol{ef}}^{\boldsymbol{2}}\boldsymbol{+}\boldsymbol{\sigma}_{\boldsymbol{\varepsilon}}^{\boldsymbol{2}}}$ |  |  |
| **KS** |  | $\boldsymbol{h}_{\boldsymbol{kin}}^{\boldsymbol{2}}\boldsymbol{=}\frac{\boldsymbol{\sigma}_{\boldsymbol{kin}}^{\boldsymbol{2}}}{\boldsymbol{\sigma}_{\boldsymbol{kin}}^{\boldsymbol{2}}\boldsymbol{+}\boldsymbol{\sigma}_{\boldsymbol{es}}^{\boldsymbol{2}}\boldsymbol{+}\boldsymbol{\sigma}_{\boldsymbol{\varepsilon}}^{\boldsymbol{2}}}$ |  | $\boldsymbol{e}_{\boldsymbol{s}}^{\boldsymbol{2}}\boldsymbol{=}\frac{\boldsymbol{\sigma}_{\boldsymbol{es}}^{\boldsymbol{2}}}{\boldsymbol{\sigma}_{\boldsymbol{kin}}^{\boldsymbol{2}}\boldsymbol{+}\boldsymbol{\sigma}_{\boldsymbol{es}}^{\boldsymbol{2}}\boldsymbol{+}\boldsymbol{\sigma}_{\boldsymbol{\varepsilon}}^{\boldsymbol{2}}}$ |  |
| **KC** |  | $\boldsymbol{h}_{\boldsymbol{kin}}^{\boldsymbol{2}}\boldsymbol{=}\frac{\boldsymbol{\sigma}_{\boldsymbol{kin}}^{\boldsymbol{2}}}{\boldsymbol{\sigma}_{\boldsymbol{kin}}^{\boldsymbol{2}}\boldsymbol{+}\boldsymbol{\sigma}_{\boldsymbol{ec}}^{\boldsymbol{2}}\boldsymbol{+}\boldsymbol{\sigma}_{\boldsymbol{\varepsilon}}^{\boldsymbol{2}}}$ |  |  | $\boldsymbol{e}_{\boldsymbol{c}}^{\boldsymbol{2}}\boldsymbol{=}\frac{\boldsymbol{\sigma}_{\boldsymbol{ec}}^{\boldsymbol{2}}}{\boldsymbol{\sigma}_{\boldsymbol{kin}}^{\boldsymbol{2}}\boldsymbol{+}\boldsymbol{\sigma}_{\boldsymbol{ec}}^{\boldsymbol{2}}\boldsymbol{+}\boldsymbol{\sigma}_{\boldsymbol{\varepsilon}}^{\boldsymbol{2}}}$ |
| **FS** |  |  | $\boldsymbol{e}_{\boldsymbol{f}}^{\boldsymbol{2}}\boldsymbol{=}\frac{\boldsymbol{\sigma}_{\boldsymbol{ef}}^{\boldsymbol{2}}}{\boldsymbol{\sigma}_{\boldsymbol{ef}}^{\boldsymbol{2}}\boldsymbol{+}{\boldsymbol{\sigma}_{\boldsymbol{es}}^{\boldsymbol{2}}\boldsymbol{+\sigma}}_{\boldsymbol{\varepsilon}}^{\boldsymbol{2}}}$ | $\boldsymbol{e}_{\boldsymbol{s}}^{\boldsymbol{2}}\boldsymbol{=}\frac{\boldsymbol{\sigma}_{\boldsymbol{es}}^{\boldsymbol{2}}}{\boldsymbol{\sigma}_{\boldsymbol{ef}}^{\boldsymbol{2}}\boldsymbol{+}{\boldsymbol{\sigma}_{\boldsymbol{es}}^{\boldsymbol{2}}\boldsymbol{+\sigma}}_{\boldsymbol{\varepsilon}}^{\boldsymbol{2}}}$ |  |
| **FC** |  |  | $\boldsymbol{e}_{\boldsymbol{f}}^{\boldsymbol{2}}\boldsymbol{=}\frac{\boldsymbol{\sigma}_{\boldsymbol{ef}}^{\boldsymbol{2}}}{\boldsymbol{\sigma}_{\boldsymbol{ef}}^{\boldsymbol{2}}\boldsymbol{+}{\boldsymbol{\sigma}_{\boldsymbol{ec}}^{\boldsymbol{2}}\boldsymbol{+\sigma}}_{\boldsymbol{\varepsilon}}^{\boldsymbol{2}}}$ |  | $\boldsymbol{e}_{\boldsymbol{c}}^{\boldsymbol{2}}\boldsymbol{=}\frac{\boldsymbol{\sigma}_{\boldsymbol{ec}}^{\boldsymbol{2}}}{\boldsymbol{\sigma}_{\boldsymbol{ef}}^{\boldsymbol{2}}\boldsymbol{+}{\boldsymbol{\sigma}_{\boldsymbol{ec}}^{\boldsymbol{2}}\boldsymbol{+\sigma}}_{\boldsymbol{\varepsilon}}^{\boldsymbol{2}}}$ |
| **SC** |  |  |  | $\boldsymbol{e}_{\boldsymbol{s}}^{\boldsymbol{2}}\boldsymbol{=}\frac{\boldsymbol{\sigma}_{\boldsymbol{es}}^{\boldsymbol{2}}}{\boldsymbol{\sigma}_{\boldsymbol{es}}^{\boldsymbol{2}}\boldsymbol{+}{\boldsymbol{\sigma}_{\boldsymbol{ec}}^{\boldsymbol{2}}\boldsymbol{+\sigma}}_{\boldsymbol{\varepsilon}}^{\boldsymbol{2}}}$ | $\boldsymbol{e}_{\boldsymbol{c}}^{\boldsymbol{2}}\boldsymbol{=}\frac{\boldsymbol{\sigma}_{\boldsymbol{ec}}^{\boldsymbol{2}}}{\boldsymbol{\sigma}_{\boldsymbol{es}}^{\boldsymbol{2}}\boldsymbol{+}{\boldsymbol{\sigma}_{\boldsymbol{ec}}^{\boldsymbol{2}}\boldsymbol{+\sigma}}_{\boldsymbol{\varepsilon}}^{\boldsymbol{2}}}$ |
| **GKF** | $\boldsymbol{h}_{\boldsymbol{g}}^{\boldsymbol{2}}\boldsymbol{=}\frac{\boldsymbol{\sigma}_{\boldsymbol{g}}^{\boldsymbol{2}}}{\boldsymbol{\sigma}_{\boldsymbol{g}}^{\boldsymbol{2}}\boldsymbol{+}\boldsymbol{\sigma}_{\boldsymbol{kin}}^{\boldsymbol{2}}\boldsymbol{+}\boldsymbol{\sigma}_{\boldsymbol{ef}}^{\boldsymbol{2}}\boldsymbol{+}\boldsymbol{\sigma}_{\boldsymbol{\varepsilon}}^{\boldsymbol{2}}}$ | $\boldsymbol{h}_{\boldsymbol{kin}}^{\boldsymbol{2}}\boldsymbol{=}\frac{\boldsymbol{\sigma}_{\boldsymbol{kin}}^{\boldsymbol{2}}}{\boldsymbol{\sigma}_{\boldsymbol{g}}^{\boldsymbol{2}}\boldsymbol{+}\boldsymbol{\sigma}_{\boldsymbol{kin}}^{\boldsymbol{2}}\boldsymbol{+}\boldsymbol{\sigma}_{\boldsymbol{ef}}^{\boldsymbol{2}}\boldsymbol{+}\boldsymbol{\sigma}_{\boldsymbol{\varepsilon}}^{\boldsymbol{2}}}$ | $\boldsymbol{e}_{\boldsymbol{f}}^{\boldsymbol{2}}\boldsymbol{=}\frac{\boldsymbol{\sigma}_{\boldsymbol{ef}}^{\boldsymbol{2}}}{\boldsymbol{\sigma}_{\boldsymbol{g}}^{\boldsymbol{2}}\boldsymbol{+}\boldsymbol{\sigma}_{\boldsymbol{kin}}^{\boldsymbol{2}}\boldsymbol{+}\boldsymbol{\sigma}_{\boldsymbol{ef}}^{\boldsymbol{2}}\boldsymbol{+}\boldsymbol{\sigma}_{\boldsymbol{\varepsilon}}^{\boldsymbol{2}}}$ |  |  |
| **GKS** | $\boldsymbol{h}_{\boldsymbol{g}}^{\boldsymbol{2}}\boldsymbol{=}\frac{\boldsymbol{\sigma}_{\boldsymbol{g}}^{\boldsymbol{2}}}{\boldsymbol{\sigma}_{\boldsymbol{g}}^{\boldsymbol{2}}\boldsymbol{+}\boldsymbol{\sigma}_{\boldsymbol{kin}}^{\boldsymbol{2}}\boldsymbol{+}\boldsymbol{\sigma}_{\boldsymbol{es}}^{\boldsymbol{2}}\boldsymbol{+}\boldsymbol{\sigma}_{\boldsymbol{\varepsilon}}^{\boldsymbol{2}}}$ | $\boldsymbol{h}_{\boldsymbol{kin}}^{\boldsymbol{2}}\boldsymbol{=}\frac{\boldsymbol{\sigma}_{\boldsymbol{kin}}^{\boldsymbol{2}}}{\boldsymbol{\sigma}_{\boldsymbol{g}}^{\boldsymbol{2}}\boldsymbol{+}\boldsymbol{\sigma}_{\boldsymbol{kin}}^{\boldsymbol{2}}\boldsymbol{+}\boldsymbol{\sigma}_{\boldsymbol{es}}^{\boldsymbol{2}}\boldsymbol{+}\boldsymbol{\sigma}_{\boldsymbol{\varepsilon}}^{\boldsymbol{2}}}$ |  | $\boldsymbol{e}_{\boldsymbol{S}}^{\boldsymbol{2}}\boldsymbol{=}\frac{\boldsymbol{\sigma}_{\boldsymbol{es}}^{\boldsymbol{2}}}{\boldsymbol{\sigma}_{\boldsymbol{g}}^{\boldsymbol{2}}\boldsymbol{+}\boldsymbol{\sigma}_{\boldsymbol{kin}}^{\boldsymbol{2}}\boldsymbol{+}\boldsymbol{\sigma}_{\boldsymbol{es}}^{\boldsymbol{2}}\boldsymbol{+}\boldsymbol{\sigma}_{\boldsymbol{\varepsilon}}^{\boldsymbol{2}}}$ |  |
| **GKC** | $\boldsymbol{h}_{\boldsymbol{g}}^{\boldsymbol{2}}\boldsymbol{=}\frac{\boldsymbol{\sigma}_{\boldsymbol{g}}^{\boldsymbol{2}}}{\boldsymbol{\sigma}_{\boldsymbol{g}}^{\boldsymbol{2}}\boldsymbol{+}\boldsymbol{\sigma}_{\boldsymbol{kin}}^{\boldsymbol{2}}\boldsymbol{+}\boldsymbol{\sigma}_{\boldsymbol{ec}}^{\boldsymbol{2}}\boldsymbol{+}\boldsymbol{\sigma}_{\boldsymbol{\varepsilon}}^{\boldsymbol{2}}}$ | $\boldsymbol{h}_{\boldsymbol{kin}}^{\boldsymbol{2}}\boldsymbol{=}\frac{\boldsymbol{\sigma}_{\boldsymbol{kin}}^{\boldsymbol{2}}}{\boldsymbol{\sigma}_{\boldsymbol{g}}^{\boldsymbol{2}}\boldsymbol{+}\boldsymbol{\sigma}_{\boldsymbol{kin}}^{\boldsymbol{2}}\boldsymbol{+}\boldsymbol{\sigma}_{\boldsymbol{ec}}^{\boldsymbol{2}}\boldsymbol{+}\boldsymbol{\sigma}_{\boldsymbol{\varepsilon}}^{\boldsymbol{2}}}$ |  |  | $\boldsymbol{e}_{\boldsymbol{c}}^{\boldsymbol{2}}\boldsymbol{=}\frac{\boldsymbol{\sigma}_{\boldsymbol{ec}}^{\boldsymbol{2}}}{\boldsymbol{\sigma}_{\boldsymbol{g}}^{\boldsymbol{2}}\boldsymbol{+}\boldsymbol{\sigma}_{\boldsymbol{gkin}}^{\boldsymbol{2}}\boldsymbol{+}\boldsymbol{\sigma}_{\boldsymbol{ec}}^{\boldsymbol{2}}\boldsymbol{+}\boldsymbol{\sigma}_{\boldsymbol{\varepsilon}}^{\boldsymbol{2}}}$ |
| **GFS** | $\boldsymbol{h}_{\boldsymbol{g}}^{\boldsymbol{2}}\boldsymbol{=}\frac{\boldsymbol{\sigma}_{\boldsymbol{g}}^{\boldsymbol{2}}}{\boldsymbol{\sigma}_{\boldsymbol{g}}^{\boldsymbol{2}}\boldsymbol{+}\boldsymbol{\sigma}_{\boldsymbol{ef}}^{\boldsymbol{2}}\boldsymbol{+}\boldsymbol{\sigma}_{\boldsymbol{es}}^{\boldsymbol{2}}\boldsymbol{+}\boldsymbol{\sigma}_{\boldsymbol{\varepsilon}}^{\boldsymbol{2}}}$ |  | $\boldsymbol{e}_{\boldsymbol{f}}^{\boldsymbol{2}}\boldsymbol{=}\frac{\boldsymbol{\sigma}_{\boldsymbol{ef}}^{\boldsymbol{2}}}{\boldsymbol{\sigma}_{\boldsymbol{g}}^{\boldsymbol{2}}\boldsymbol{+}\boldsymbol{\sigma}_{\boldsymbol{ef}}^{\boldsymbol{2}}\boldsymbol{+}\boldsymbol{\sigma}_{\boldsymbol{es}}^{\boldsymbol{2}}\boldsymbol{+}\boldsymbol{\sigma}_{\boldsymbol{\varepsilon}}^{\boldsymbol{2}}}$ | $\boldsymbol{e}_{\boldsymbol{s}}^{\boldsymbol{2}}\boldsymbol{=}\frac{\boldsymbol{\sigma}_{\boldsymbol{es}}^{\boldsymbol{2}}}{\boldsymbol{\sigma}_{\boldsymbol{g}}^{\boldsymbol{2}}\boldsymbol{+}\boldsymbol{\sigma}_{\boldsymbol{ef}}^{\boldsymbol{2}}\boldsymbol{+}\boldsymbol{\sigma}_{\boldsymbol{es}}^{\boldsymbol{2}}\boldsymbol{+}\boldsymbol{\sigma}_{\boldsymbol{\varepsilon}}^{\boldsymbol{2}}}$ |  |
| **GFC** | $\boldsymbol{h}_{\boldsymbol{g}}^{\boldsymbol{2}}\boldsymbol{=}\frac{\boldsymbol{\sigma}_{\boldsymbol{g}}^{\boldsymbol{2}}}{\boldsymbol{\sigma}_{\boldsymbol{g}}^{\boldsymbol{2}}\boldsymbol{+}\boldsymbol{\sigma}_{\boldsymbol{ef}}^{\boldsymbol{2}}\boldsymbol{+}\boldsymbol{\sigma}_{\boldsymbol{ec}}^{\boldsymbol{2}}\boldsymbol{+}\boldsymbol{\sigma}_{\boldsymbol{\varepsilon}}^{\boldsymbol{2}}}$ |  | $\boldsymbol{e}_{\boldsymbol{f}}^{\boldsymbol{2}}\boldsymbol{=}\frac{\boldsymbol{\sigma}_{\boldsymbol{ef}}^{\boldsymbol{2}}}{\boldsymbol{\sigma}_{\boldsymbol{g}}^{\boldsymbol{2}}\boldsymbol{+}\boldsymbol{\sigma}_{\boldsymbol{ef}}^{\boldsymbol{2}}\boldsymbol{+}\boldsymbol{\sigma}_{\boldsymbol{ec}}^{\boldsymbol{2}}\boldsymbol{+}\boldsymbol{\sigma}_{\boldsymbol{\varepsilon}}^{\boldsymbol{2}}}$ |  | $\boldsymbol{e}_{\boldsymbol{c}}^{\boldsymbol{2}}\boldsymbol{=}\frac{\boldsymbol{\sigma}_{\boldsymbol{ec}}^{\boldsymbol{2}}}{\boldsymbol{\sigma}_{\boldsymbol{g}}^{\boldsymbol{2}}\boldsymbol{+}\boldsymbol{\sigma}_{\boldsymbol{ef}}^{\boldsymbol{2}}\boldsymbol{+}\boldsymbol{\sigma}_{\boldsymbol{ec}}^{\boldsymbol{2}}\boldsymbol{+}\boldsymbol{\sigma}_{\boldsymbol{\varepsilon}}^{\boldsymbol{2}}}$ |
| **GSC** | $\boldsymbol{h}_{\boldsymbol{g}}^{\boldsymbol{2}}\boldsymbol{=}\frac{\boldsymbol{\sigma}_{\boldsymbol{g}}^{\boldsymbol{2}}}{\boldsymbol{\sigma}_{\boldsymbol{g}}^{\boldsymbol{2}}\boldsymbol{+}\boldsymbol{\sigma}_{\boldsymbol{es}}^{\boldsymbol{2}}\boldsymbol{+}\boldsymbol{\sigma}_{\boldsymbol{ec}}^{\boldsymbol{2}}\boldsymbol{+}\boldsymbol{\sigma}_{\boldsymbol{\varepsilon}}^{\boldsymbol{2}}}$ |  |  | $\boldsymbol{e}_{\boldsymbol{s}}^{\boldsymbol{2}}\boldsymbol{=}\frac{\boldsymbol{\sigma}_{\boldsymbol{es}}^{\boldsymbol{2}}}{\boldsymbol{\sigma}_{\boldsymbol{g}}^{\boldsymbol{2}}\boldsymbol{+}\boldsymbol{\sigma}_{\boldsymbol{es}}^{\boldsymbol{2}}\boldsymbol{+}\boldsymbol{\sigma}_{\boldsymbol{ec}}^{\boldsymbol{2}}\boldsymbol{+}\boldsymbol{\sigma}_{\boldsymbol{\varepsilon}}^{\boldsymbol{2}}}$ | $\boldsymbol{e}_{\boldsymbol{c}}^{\boldsymbol{2}}\boldsymbol{=}\frac{\boldsymbol{\sigma}_{\boldsymbol{ec}}^{\boldsymbol{2}}}{\boldsymbol{\sigma}_{\boldsymbol{g}}^{\boldsymbol{2}}\boldsymbol{+}\boldsymbol{\sigma}_{\boldsymbol{es}}^{\boldsymbol{2}}\boldsymbol{+}\boldsymbol{\sigma}_{\boldsymbol{ec}}^{\boldsymbol{2}}\boldsymbol{+}\boldsymbol{\sigma}_{\boldsymbol{\varepsilon}}^{\boldsymbol{2}}}$ |
| **KFS** |  | $\boldsymbol{h}_{\boldsymbol{kin}}^{\boldsymbol{2}}\boldsymbol{=}\frac{\boldsymbol{\sigma}_{\boldsymbol{kin}}^{\boldsymbol{2}}}{\boldsymbol{\sigma}_{\boldsymbol{kin}}^{\boldsymbol{2}}\boldsymbol{+}\boldsymbol{\sigma}_{\boldsymbol{ef}}^{\boldsymbol{2}}\boldsymbol{+}\boldsymbol{\sigma}_{\boldsymbol{es}}^{\boldsymbol{2}}\boldsymbol{+}\boldsymbol{\sigma}_{\boldsymbol{\varepsilon}}^{\boldsymbol{2}}}$ | $\boldsymbol{e}_{\boldsymbol{f}}^{\boldsymbol{2}}\boldsymbol{=}\frac{\boldsymbol{\sigma}_{\boldsymbol{ef}}^{\boldsymbol{2}}}{\boldsymbol{\sigma}_{\boldsymbol{kin}}^{\boldsymbol{2}}\boldsymbol{+}\boldsymbol{\sigma}_{\boldsymbol{ef}}^{\boldsymbol{2}}\boldsymbol{+}\boldsymbol{\sigma}_{\boldsymbol{es}}^{\boldsymbol{2}}\boldsymbol{+}\boldsymbol{\sigma}_{\boldsymbol{\varepsilon}}^{\boldsymbol{2}}}$ | $\boldsymbol{e}_{\boldsymbol{s}}^{\boldsymbol{2}}\boldsymbol{=}\frac{\boldsymbol{\sigma}_{\boldsymbol{es}}^{\boldsymbol{2}}}{\boldsymbol{\sigma}_{\boldsymbol{kin}}^{\boldsymbol{2}}\boldsymbol{+}\boldsymbol{\sigma}_{\boldsymbol{ef}}^{\boldsymbol{2}}\boldsymbol{+}\boldsymbol{\sigma}_{\boldsymbol{es}}^{\boldsymbol{2}}\boldsymbol{+}\boldsymbol{\sigma}_{\boldsymbol{\varepsilon}}^{\boldsymbol{2}}}$ |  |
| **KFC** |  | $\boldsymbol{h}_{\boldsymbol{kin}}^{\boldsymbol{2}}\boldsymbol{=}\frac{\boldsymbol{\sigma}_{\boldsymbol{kin}}^{\boldsymbol{2}}}{\boldsymbol{\sigma}_{\boldsymbol{kin}}^{\boldsymbol{2}}\boldsymbol{+}\boldsymbol{\sigma}_{\boldsymbol{ef}}^{\boldsymbol{2}}\boldsymbol{+}\boldsymbol{\sigma}_{\boldsymbol{ec}}^{\boldsymbol{2}}\boldsymbol{+}\boldsymbol{\sigma}_{\boldsymbol{\varepsilon}}^{\boldsymbol{2}}}$ | $\boldsymbol{e}_{\boldsymbol{f}}^{\boldsymbol{2}}\boldsymbol{=}\frac{\boldsymbol{\sigma}_{\boldsymbol{ef}}^{\boldsymbol{2}}}{\boldsymbol{\sigma}_{\boldsymbol{kin}}^{\boldsymbol{2}}\boldsymbol{+}\boldsymbol{\sigma}_{\boldsymbol{ef}}^{\boldsymbol{2}}\boldsymbol{+}\boldsymbol{\sigma}_{\boldsymbol{ec}}^{\boldsymbol{2}}\boldsymbol{+}\boldsymbol{\sigma}_{\boldsymbol{\varepsilon}}^{\boldsymbol{2}}}$ |  | $\boldsymbol{e}_{\boldsymbol{c}}^{\boldsymbol{2}}\boldsymbol{=}\frac{\boldsymbol{\sigma}_{\boldsymbol{ec}}^{\boldsymbol{2}}}{\boldsymbol{\sigma}_{\boldsymbol{kin}}^{\boldsymbol{2}}\boldsymbol{+}\boldsymbol{\sigma}_{\boldsymbol{ef}}^{\boldsymbol{2}}\boldsymbol{+}\boldsymbol{\sigma}_{\boldsymbol{ec}}^{\boldsymbol{2}}\boldsymbol{+}\boldsymbol{\sigma}_{\boldsymbol{\varepsilon}}^{\boldsymbol{2}}}$ |
| **KSC** |  | $\boldsymbol{h}_{\boldsymbol{kin}}^{\boldsymbol{2}}\boldsymbol{=}\frac{\boldsymbol{\sigma}_{\boldsymbol{kin}}^{\boldsymbol{2}}}{\boldsymbol{\sigma}_{\boldsymbol{kin}}^{\boldsymbol{2}}\boldsymbol{+}\boldsymbol{\sigma}_{\boldsymbol{es}}^{\boldsymbol{2}}\boldsymbol{+}\boldsymbol{\sigma}_{\boldsymbol{ec}}^{\boldsymbol{2}}\boldsymbol{+}\boldsymbol{\sigma}_{\boldsymbol{\varepsilon}}^{\boldsymbol{2}}}$ |  | $\boldsymbol{e}_{\boldsymbol{s}}^{\boldsymbol{2}}\boldsymbol{=}\frac{\boldsymbol{\sigma}_{\boldsymbol{es}}^{\boldsymbol{2}}}{\boldsymbol{\sigma}_{\boldsymbol{kin}}^{\boldsymbol{2}}\boldsymbol{+}\boldsymbol{\sigma}_{\boldsymbol{es}}^{\boldsymbol{2}}\boldsymbol{+}\boldsymbol{\sigma}_{\boldsymbol{ec}}^{\boldsymbol{2}}\boldsymbol{+}\boldsymbol{\sigma}_{\boldsymbol{\varepsilon}}^{\boldsymbol{2}}}$ | $\boldsymbol{e}_{\boldsymbol{c}}^{\boldsymbol{2}}\boldsymbol{=}\frac{\boldsymbol{\sigma}_{\boldsymbol{ec}}^{\boldsymbol{2}}}{\boldsymbol{\sigma}_{\boldsymbol{kin}}^{\boldsymbol{2}}\boldsymbol{+}\boldsymbol{\sigma}_{\boldsymbol{es}}^{\boldsymbol{2}}\boldsymbol{+}\boldsymbol{\sigma}_{\boldsymbol{ec}}^{\boldsymbol{2}}\boldsymbol{+}\boldsymbol{\sigma}_{\boldsymbol{\varepsilon}}^{\boldsymbol{2}}}$ |
| **FSC** |  |  | $\boldsymbol{e}_{\boldsymbol{f}}^{\boldsymbol{2}}\boldsymbol{=}\frac{\boldsymbol{\sigma}_{\boldsymbol{ef}}^{\boldsymbol{2}}}{\boldsymbol{\sigma}_{\boldsymbol{ef}}^{\boldsymbol{2}}\boldsymbol{+}\boldsymbol{\sigma}_{\boldsymbol{es}}^{\boldsymbol{2}}\boldsymbol{+}\boldsymbol{\sigma}_{\boldsymbol{ec}}^{\boldsymbol{2}}\boldsymbol{+}\boldsymbol{\sigma}_{\boldsymbol{\varepsilon}}^{\boldsymbol{2}}}$ | $\boldsymbol{e}_{\boldsymbol{s}}^{\boldsymbol{2}}\boldsymbol{=}\frac{\boldsymbol{\sigma}_{\boldsymbol{es}}^{\boldsymbol{2}}}{\boldsymbol{\sigma}_{\boldsymbol{ef}}^{\boldsymbol{2}}\boldsymbol{+}\boldsymbol{\sigma}_{\boldsymbol{es}}^{\boldsymbol{2}}\boldsymbol{+}\boldsymbol{\sigma}_{\boldsymbol{ec}}^{\boldsymbol{2}}\boldsymbol{+}\boldsymbol{\sigma}_{\boldsymbol{\varepsilon}}^{\boldsymbol{2}}}$ | $\boldsymbol{e}_{\boldsymbol{c}}^{\boldsymbol{2}}\boldsymbol{=}\frac{\boldsymbol{\sigma}_{\boldsymbol{ec}}^{\boldsymbol{2}}}{\boldsymbol{\sigma}_{\boldsymbol{ef}}^{\boldsymbol{2}}\boldsymbol{+}\boldsymbol{\sigma}_{\boldsymbol{es}}^{\boldsymbol{2}}\boldsymbol{+}\boldsymbol{\sigma}_{\boldsymbol{ec}}^{\boldsymbol{2}}\boldsymbol{+}\boldsymbol{\sigma}_{\boldsymbol{\varepsilon}}^{\boldsymbol{2}}}$ |
| **GKFS** | $\boldsymbol{h}_{\boldsymbol{g}}^{\boldsymbol{2}}\boldsymbol{=}\frac{\boldsymbol{\sigma}_{\boldsymbol{g}}^{\boldsymbol{2}}}{\boldsymbol{\sigma}_{\boldsymbol{g}}^{\boldsymbol{2}}\boldsymbol{+}{\boldsymbol{\sigma}_{\boldsymbol{kin}}^{\boldsymbol{2}}\boldsymbol{+\sigma}}_{\boldsymbol{ef}}^{\boldsymbol{2}}\boldsymbol{+}\boldsymbol{\sigma}_{\boldsymbol{es}}^{\boldsymbol{2}}\boldsymbol{+}\boldsymbol{\sigma}_{\boldsymbol{\varepsilon}}^{\boldsymbol{2}}}$ | $\boldsymbol{h}_{\boldsymbol{kin}}^{\boldsymbol{2}}\boldsymbol{=}\frac{\boldsymbol{\sigma}_{\boldsymbol{kin}}^{\boldsymbol{2}}}{\boldsymbol{\sigma}_{\boldsymbol{g}}^{\boldsymbol{2}}\boldsymbol{+}{\boldsymbol{\sigma}_{\boldsymbol{kin}}^{\boldsymbol{2}}\boldsymbol{+\sigma}}_{\boldsymbol{ef}}^{\boldsymbol{2}}\boldsymbol{+}\boldsymbol{\sigma}_{\boldsymbol{es}}^{\boldsymbol{2}}\boldsymbol{+}\boldsymbol{\sigma}_{\boldsymbol{\varepsilon}}^{\boldsymbol{2}}}$ | $\boldsymbol{e}_{\boldsymbol{f}}^{\boldsymbol{2}}\boldsymbol{=}\frac{\boldsymbol{\sigma}_{\boldsymbol{ef}}^{\boldsymbol{2}}}{\boldsymbol{\sigma}_{\boldsymbol{g}}^{\boldsymbol{2}}\boldsymbol{+}{\boldsymbol{\sigma}_{\boldsymbol{kin}}^{\boldsymbol{2}}\boldsymbol{+\sigma}}_{\boldsymbol{ef}}^{\boldsymbol{2}}\boldsymbol{+}\boldsymbol{\sigma}_{\boldsymbol{es}}^{\boldsymbol{2}}\boldsymbol{+}\boldsymbol{\sigma}_{\boldsymbol{\varepsilon}}^{\boldsymbol{2}}}$ | $\boldsymbol{e}_{\boldsymbol{s}}^{\boldsymbol{2}}\boldsymbol{=}\frac{\boldsymbol{\sigma}_{\boldsymbol{es}}^{\boldsymbol{2}}}{\boldsymbol{\sigma}_{\boldsymbol{g}}^{\boldsymbol{2}}\boldsymbol{+}{\boldsymbol{\sigma}_{\boldsymbol{kin}}^{\boldsymbol{2}}\boldsymbol{+\sigma}}_{\boldsymbol{ef}}^{\boldsymbol{2}}\boldsymbol{+}\boldsymbol{\sigma}_{\boldsymbol{es}}^{\boldsymbol{2}}\boldsymbol{+}\boldsymbol{\sigma}_{\boldsymbol{\varepsilon}}^{\boldsymbol{2}}}$ |  |
| **GKFC** | $\boldsymbol{h}_{\boldsymbol{g}}^{\boldsymbol{2}}\boldsymbol{=}\frac{\boldsymbol{\sigma}_{\boldsymbol{g}}^{\boldsymbol{2}}}{\boldsymbol{\sigma}_{\boldsymbol{g}}^{\boldsymbol{2}}\boldsymbol{+}{\boldsymbol{\sigma}_{\boldsymbol{kin}}^{\boldsymbol{2}}\boldsymbol{+\sigma}}_{\boldsymbol{ef}}^{\boldsymbol{2}}\boldsymbol{+}\boldsymbol{\sigma}_{\boldsymbol{ec}}^{\boldsymbol{2}}\boldsymbol{+}\boldsymbol{\sigma}_{\boldsymbol{\varepsilon}}^{\boldsymbol{2}}}$ | $\boldsymbol{h}_{\boldsymbol{kin}}^{\boldsymbol{2}}\boldsymbol{=}\frac{\boldsymbol{\sigma}_{\boldsymbol{kin}}^{\boldsymbol{2}}}{\boldsymbol{\sigma}_{\boldsymbol{g}}^{\boldsymbol{2}}\boldsymbol{+}{\boldsymbol{\sigma}_{\boldsymbol{kin}}^{\boldsymbol{2}}\boldsymbol{+\sigma}}_{\boldsymbol{ef}}^{\boldsymbol{2}}\boldsymbol{+}\boldsymbol{\sigma}_{\boldsymbol{ec}}^{\boldsymbol{2}}\boldsymbol{+}\boldsymbol{\sigma}_{\boldsymbol{\varepsilon}}^{\boldsymbol{2}}}$ | $\boldsymbol{e}_{\boldsymbol{f}}^{\boldsymbol{2}}\boldsymbol{=}\frac{\boldsymbol{\sigma}_{\boldsymbol{ef}}^{\boldsymbol{2}}}{\boldsymbol{\sigma}_{\boldsymbol{g}}^{\boldsymbol{2}}\boldsymbol{+}{\boldsymbol{\sigma}_{\boldsymbol{kin}}^{\boldsymbol{2}}\boldsymbol{+\sigma}}_{\boldsymbol{ef}}^{\boldsymbol{2}}\boldsymbol{+}\boldsymbol{\sigma}_{\boldsymbol{ec}}^{\boldsymbol{2}}\boldsymbol{+}\boldsymbol{\sigma}_{\boldsymbol{\varepsilon}}^{\boldsymbol{2}}}$ |  | $\boldsymbol{e}_{\boldsymbol{c}}^{\boldsymbol{2}}\boldsymbol{=}\frac{\boldsymbol{\sigma}_{\boldsymbol{ec}}^{\boldsymbol{2}}}{\boldsymbol{\sigma}_{\boldsymbol{g}}^{\boldsymbol{2}}\boldsymbol{+}{\boldsymbol{\sigma}_{\boldsymbol{kin}}^{\boldsymbol{2}}\boldsymbol{+\sigma}}_{\boldsymbol{ef}}^{\boldsymbol{2}}\boldsymbol{+}\boldsymbol{\sigma}_{\boldsymbol{ec}}^{\boldsymbol{2}}\boldsymbol{+}\boldsymbol{\sigma}_{\boldsymbol{\varepsilon}}^{\boldsymbol{2}}}$ |
| **GKSC** | $\boldsymbol{h}_{\boldsymbol{g}}^{\boldsymbol{2}}\boldsymbol{=}\frac{\boldsymbol{\sigma}_{\boldsymbol{g}}^{\boldsymbol{2}}}{\boldsymbol{\sigma}_{\boldsymbol{g}}^{\boldsymbol{2}}\boldsymbol{+}{\boldsymbol{\sigma}_{\boldsymbol{kin}}^{\boldsymbol{2}}\boldsymbol{+\sigma}}_{\boldsymbol{es}}^{\boldsymbol{2}}\boldsymbol{+}\boldsymbol{\sigma}_{\boldsymbol{ec}}^{\boldsymbol{2}}\boldsymbol{+}\boldsymbol{\sigma}_{\boldsymbol{\varepsilon}}^{\boldsymbol{2}}}$ | $\boldsymbol{h}_{\boldsymbol{kin}}^{\boldsymbol{2}}\boldsymbol{=}\frac{\boldsymbol{\sigma}_{\boldsymbol{kin}}^{\boldsymbol{2}}}{\boldsymbol{\sigma}_{\boldsymbol{g}}^{\boldsymbol{2}}\boldsymbol{+}{\boldsymbol{\sigma}_{\boldsymbol{kin}}^{\boldsymbol{2}}\boldsymbol{+\sigma}}_{\boldsymbol{es}}^{\boldsymbol{2}}\boldsymbol{+}\boldsymbol{\sigma}_{\boldsymbol{ec}}^{\boldsymbol{2}}\boldsymbol{+}\boldsymbol{\sigma}_{\boldsymbol{\varepsilon}}^{\boldsymbol{2}}}$ |  | $\boldsymbol{e}_{\boldsymbol{s}}^{\boldsymbol{2}}\boldsymbol{=}\frac{\boldsymbol{\sigma}_{\boldsymbol{es}}^{\boldsymbol{2}}}{\boldsymbol{\sigma}_{\boldsymbol{g}}^{\boldsymbol{2}}\boldsymbol{+}{\boldsymbol{\sigma}_{\boldsymbol{kin}}^{\boldsymbol{2}}\boldsymbol{+\sigma}}_{\boldsymbol{es}}^{\boldsymbol{2}}\boldsymbol{+}\boldsymbol{\sigma}_{\boldsymbol{ec}}^{\boldsymbol{2}}\boldsymbol{+}\boldsymbol{\sigma}_{\boldsymbol{\varepsilon}}^{\boldsymbol{2}}}$ | $\boldsymbol{e}_{\boldsymbol{c}}^{\boldsymbol{2}}\boldsymbol{=}\frac{\boldsymbol{\sigma}_{\boldsymbol{ec}}^{\boldsymbol{2}}}{\boldsymbol{\sigma}_{\boldsymbol{g}}^{\boldsymbol{2}}\boldsymbol{+}{\boldsymbol{\sigma}_{\boldsymbol{kin}}^{\boldsymbol{2}}\boldsymbol{+\sigma}}_{\boldsymbol{es}}^{\boldsymbol{2}}\boldsymbol{+}\boldsymbol{\sigma}_{\boldsymbol{ec}}^{\boldsymbol{2}}\boldsymbol{+}\boldsymbol{\sigma}_{\boldsymbol{\varepsilon}}^{\boldsymbol{2}}}$ |
| **GFSC** | $\boldsymbol{h}_{\boldsymbol{g}}^{\boldsymbol{2}}\boldsymbol{=}\frac{\boldsymbol{\sigma}_{\boldsymbol{g}}^{\boldsymbol{2}}}{\boldsymbol{\sigma}_{\boldsymbol{g}}^{\boldsymbol{2}}\boldsymbol{+}{\boldsymbol{\sigma}_{\boldsymbol{ef}}^{\boldsymbol{2}}\boldsymbol{+\sigma}}_{\boldsymbol{es}}^{\boldsymbol{2}}\boldsymbol{+}\boldsymbol{\sigma}_{\boldsymbol{ec}}^{\boldsymbol{2}}\boldsymbol{+}\boldsymbol{\sigma}_{\boldsymbol{\varepsilon}}^{\boldsymbol{2}}}$ |  | $\boldsymbol{e}_{\boldsymbol{f}}^{\boldsymbol{2}}\boldsymbol{=}\frac{\boldsymbol{\sigma}_{\boldsymbol{ef}}^{\boldsymbol{2}}}{\boldsymbol{\sigma}_{\boldsymbol{g}}^{\boldsymbol{2}}\boldsymbol{+}{\boldsymbol{\sigma}_{\boldsymbol{ef}}^{\boldsymbol{2}}\boldsymbol{+\sigma}}_{\boldsymbol{es}}^{\boldsymbol{2}}\boldsymbol{+}\boldsymbol{\sigma}_{\boldsymbol{ec}}^{\boldsymbol{2}}\boldsymbol{+}\boldsymbol{\sigma}_{\boldsymbol{\varepsilon}}^{\boldsymbol{2}}}$ | $\boldsymbol{e}_{\boldsymbol{s}}^{\boldsymbol{2}}\boldsymbol{=}\frac{\boldsymbol{\sigma}_{\boldsymbol{es}}^{\boldsymbol{2}}}{\boldsymbol{\sigma}_{\boldsymbol{g}}^{\boldsymbol{2}}\boldsymbol{+}{\boldsymbol{\sigma}_{\boldsymbol{ef}}^{\boldsymbol{2}}\boldsymbol{+\sigma}}_{\boldsymbol{es}}^{\boldsymbol{2}}\boldsymbol{+}\boldsymbol{\sigma}_{\boldsymbol{ec}}^{\boldsymbol{2}}\boldsymbol{+}\boldsymbol{\sigma}_{\boldsymbol{\varepsilon}}^{\boldsymbol{2}}}$ | $\boldsymbol{e}_{\boldsymbol{c}}^{\boldsymbol{2}}\boldsymbol{=}\frac{\boldsymbol{\sigma}_{\boldsymbol{ec}}^{\boldsymbol{2}}}{\boldsymbol{\sigma}_{\boldsymbol{g}}^{\boldsymbol{2}}\boldsymbol{+}{\boldsymbol{\sigma}_{\boldsymbol{ef}}^{\boldsymbol{2}}\boldsymbol{+\sigma}}_{\boldsymbol{es}}^{\boldsymbol{2}}\boldsymbol{+}\boldsymbol{\sigma}_{\boldsymbol{ec}}^{\boldsymbol{2}}\boldsymbol{+}\boldsymbol{\sigma}_{\boldsymbol{\varepsilon}}^{\boldsymbol{2}}}$ |
| **KFSC** |  | $\boldsymbol{h}_{\boldsymbol{kin}}^{\boldsymbol{2}}\boldsymbol{=}\frac{\boldsymbol{\sigma}_{\boldsymbol{kin}}^{\boldsymbol{2}}}{\boldsymbol{\sigma}_{\boldsymbol{kin}}^{\boldsymbol{2}}\boldsymbol{+}{\boldsymbol{\sigma}_{\boldsymbol{ef}}^{\boldsymbol{2}}\boldsymbol{+\sigma}}_{\boldsymbol{es}}^{\boldsymbol{2}}\boldsymbol{+}\boldsymbol{\sigma}_{\boldsymbol{ec}}^{\boldsymbol{2}}\boldsymbol{+}\boldsymbol{\sigma}_{\boldsymbol{\varepsilon}}^{\boldsymbol{2}}}$ | $\boldsymbol{e}_{\boldsymbol{f}}^{\boldsymbol{2}}\boldsymbol{=}\frac{\boldsymbol{\sigma}_{\boldsymbol{ef}}^{\boldsymbol{2}}}{\boldsymbol{\sigma}_{\boldsymbol{kin}}^{\boldsymbol{2}}\boldsymbol{+}{\boldsymbol{\sigma}_{\boldsymbol{ef}}^{\boldsymbol{2}}\boldsymbol{+\sigma}}_{\boldsymbol{es}}^{\boldsymbol{2}}\boldsymbol{+}\boldsymbol{\sigma}_{\boldsymbol{ec}}^{\boldsymbol{2}}\boldsymbol{+}\boldsymbol{\sigma}_{\boldsymbol{\varepsilon}}^{\boldsymbol{2}}}$ | $\boldsymbol{e}_{\boldsymbol{s}}^{\boldsymbol{2}}\boldsymbol{=}\frac{\boldsymbol{\sigma}_{\boldsymbol{es}}^{\boldsymbol{2}}}{\boldsymbol{\sigma}_{\boldsymbol{kin}}^{\boldsymbol{2}}\boldsymbol{+}{\boldsymbol{\sigma}_{\boldsymbol{ef}}^{\boldsymbol{2}}\boldsymbol{+\sigma}}_{\boldsymbol{es}}^{\boldsymbol{2}}\boldsymbol{+}\boldsymbol{\sigma}_{\boldsymbol{ec}}^{\boldsymbol{2}}\boldsymbol{+}\boldsymbol{\sigma}_{\boldsymbol{\varepsilon}}^{\boldsymbol{2}}}$ | $\boldsymbol{e}_{\boldsymbol{c}}^{\boldsymbol{2}}\boldsymbol{=}\frac{\boldsymbol{\sigma}_{\boldsymbol{ec}}^{\boldsymbol{2}}}{\boldsymbol{\sigma}_{\boldsymbol{kin}}^{\boldsymbol{2}}\boldsymbol{+}{\boldsymbol{\sigma}_{\boldsymbol{ef}}^{\boldsymbol{2}}\boldsymbol{+\sigma}}_{\boldsymbol{es}}^{\boldsymbol{2}}\boldsymbol{+}\boldsymbol{\sigma}_{\boldsymbol{ec}}^{\boldsymbol{2}}\boldsymbol{+}\boldsymbol{\sigma}_{\boldsymbol{\varepsilon}}^{\boldsymbol{2}}}$ |
| **GKFSC** | $\boldsymbol{h}_{\boldsymbol{g}}^{\boldsymbol{2}}\boldsymbol{=}\frac{\boldsymbol{\sigma}_{\boldsymbol{g}}^{\boldsymbol{2}}}{\boldsymbol{\sigma}_{\boldsymbol{g}}^{\boldsymbol{2}}\boldsymbol{+}{\boldsymbol{\sigma}_{\boldsymbol{kin}}^{\boldsymbol{2}}\boldsymbol{+}\boldsymbol{\sigma}_{\boldsymbol{ef}}^{\boldsymbol{2}}\boldsymbol{+\sigma}}_{\boldsymbol{es}}^{\boldsymbol{2}}\boldsymbol{+}\boldsymbol{\sigma}_{\boldsymbol{ec}}^{\boldsymbol{2}}\boldsymbol{+}\boldsymbol{\sigma}_{\boldsymbol{\varepsilon}}^{\boldsymbol{2}}}$ | $\boldsymbol{h}_{\boldsymbol{kin}}^{\boldsymbol{2}}\boldsymbol{=}\frac{\boldsymbol{\sigma}_{\boldsymbol{kin}}^{\boldsymbol{2}}}{\boldsymbol{\sigma}_{\boldsymbol{g}}^{\boldsymbol{2}}\boldsymbol{+}{\boldsymbol{\sigma}_{\boldsymbol{kin}}^{\boldsymbol{2}}\boldsymbol{+}\boldsymbol{\sigma}_{\boldsymbol{ef}}^{\boldsymbol{2}}\boldsymbol{+\sigma}}_{\boldsymbol{es}}^{\boldsymbol{2}}\boldsymbol{+}\boldsymbol{\sigma}_{\boldsymbol{ec}}^{\boldsymbol{2}}\boldsymbol{+}\boldsymbol{\sigma}_{\boldsymbol{\varepsilon}}^{\boldsymbol{2}}}$ | $\boldsymbol{e}_{\boldsymbol{f}}^{\boldsymbol{2}}\boldsymbol{=}\frac{\boldsymbol{\sigma}_{\boldsymbol{ef}}^{\boldsymbol{2}}}{\boldsymbol{\sigma}_{\boldsymbol{g}}^{\boldsymbol{2}}\boldsymbol{+}{\boldsymbol{\sigma}_{\boldsymbol{kin}}^{\boldsymbol{2}}\boldsymbol{+}\boldsymbol{\sigma}_{\boldsymbol{ef}}^{\boldsymbol{2}}\boldsymbol{+\sigma}}_{\boldsymbol{es}}^{\boldsymbol{2}}\boldsymbol{+}\boldsymbol{\sigma}_{\boldsymbol{ec}}^{\boldsymbol{2}}\boldsymbol{+}\boldsymbol{\sigma}_{\boldsymbol{\varepsilon}}^{\boldsymbol{2}}}$ | $\boldsymbol{e}_{\boldsymbol{s}}^{\boldsymbol{2}}\boldsymbol{=}\frac{\boldsymbol{\sigma}_{\boldsymbol{es}}^{\boldsymbol{2}}}{\boldsymbol{\sigma}_{\boldsymbol{g}}^{\boldsymbol{2}}\boldsymbol{+}{\boldsymbol{\sigma}_{\boldsymbol{kin}}^{\boldsymbol{2}}\boldsymbol{+}\boldsymbol{\sigma}_{\boldsymbol{ef}}^{\boldsymbol{2}}\boldsymbol{+\sigma}}_{\boldsymbol{es}}^{\boldsymbol{2}}\boldsymbol{+}\boldsymbol{\sigma}_{\boldsymbol{ec}}^{\boldsymbol{2}}\boldsymbol{+}\boldsymbol{\sigma}_{\boldsymbol{\varepsilon}}^{\boldsymbol{2}}}$ | $\boldsymbol{e}_{\boldsymbol{c}}^{\boldsymbol{2}}\boldsymbol{=}\frac{\boldsymbol{\sigma}_{\boldsymbol{ec}}^{\boldsymbol{2}}}{\boldsymbol{\sigma}_{\boldsymbol{g}}^{\boldsymbol{2}}\boldsymbol{+}{\boldsymbol{\sigma}_{\boldsymbol{kin}}^{\boldsymbol{2}}\boldsymbol{+}\boldsymbol{\sigma}_{\boldsymbol{ef}}^{\boldsymbol{2}}\boldsymbol{+\sigma}}_{\boldsymbol{es}}^{\boldsymbol{2}}\boldsymbol{+}\boldsymbol{\sigma}_{\boldsymbol{ec}}^{\boldsymbol{2}}\boldsymbol{+}\boldsymbol{\sigma}_{\boldsymbol{\varepsilon}}^{\boldsymbol{2}}}$ |
|  | | | | | |
